# Compromised Astrocyte Swelling/Volume Regulation in the Hippocampus of the Triple Transgenic Mouse Model of Alzheimer’s Disease

**DOI:** 10.3389/fnagi.2021.783120

**Published:** 2022-01-27

**Authors:** Jana Tureckova, Monika Kamenicka, Denisa Kolenicova, Tereza Filipi, Zuzana Hermanova, Jan Kriska, Lenka Meszarosova, Barbora Pukajova, Lukas Valihrach, Peter Androvic, Daniel Zucha, Martina Chmelova, Lydia Vargova, Miroslava Anderova

**Affiliations:** ^1^Department of Cellular Neurophysiology, Institute of Experimental Medicine, Czech Academy of Sciences, Prague, Czechia; ^2^Second Faculty of Medicine, Charles University, Prague, Czechia; ^3^Laboratory of Gene Expression, Institute of Biotechnology, Czech Academy of Sciences, Vestec, Czechia; ^4^Faculty of Chemical Technology, University of Chemistry and Technology, Prague, Czechia

**Keywords:** astrocytes, ECS diffusion, volume changes, astrocyte heterogeneity, ion uptake, Alzheimer’s disease

## Abstract

In this study, we aimed to disclose the impact of amyloid-β toxicity and tau pathology on astrocyte swelling, their volume recovery and extracellular space (ECS) diffusion parameters, namely volume fraction (α) and tortuosity (λ), in a triple transgenic mouse model of Alzheimer’s disease (3xTg-AD). Astrocyte volume changes, which reflect astrocyte ability to take up ions/neurotransmitters, were quantified during and after exposure to hypo-osmotic stress, or hyperkalemia in acute hippocampal slices, and were correlated with alterations in ECS diffusion parameters. Astrocyte volume and ECS diffusion parameters were monitored during physiological aging (controls) and during AD progression in 3-, 9-, 12- and 18-month-old mice. In the hippocampus of controls α gradually declined with age, while it remained unaffected in 3xTg-AD mice during the entire time course. Moreover, age-related increases in λ occurred much earlier in 3xTg-AD animals than in controls. In 3xTg-AD mice changes in α induced by hypo-osmotic stress or hyperkalemia were comparable to those observed in controls, however, AD progression affected α recovery following exposure to both. Compared to controls, a smaller astrocyte swelling was detected in 3xTg-AD mice only during hyperkalemia. Since we observed a large variance in astrocyte swelling/volume regulation, we divided them into high- (HRA) and low-responding astrocytes (LRA). In response to hyperkalemia, the incidence of LRA was higher in 3xTg-AD mice than in controls, which may also reflect compromised K^+^ and neurotransmitter uptake. Furthermore, we performed single-cell RT-qPCR to identify possible age-related alterations in astrocytic gene expression profiles. Already in 3-month-old 3xTg-AD mice, we detected a downregulation of genes affecting the ion/neurotransmitter uptake and cell volume regulation, namely genes of glutamate transporters, α2β2 subunit of Na^+^/K^+^-ATPase, connexin 30 or Kir4.1 channel. In conclusion, the aged hippocampus of 3xTg-AD mice displays an enlarged ECS volume fraction and an increased number of obstacles, which emerge earlier than in physiological aging. Both these changes may strongly affect intercellular communication and influence astrocyte ionic/neurotransmitter uptake, which becomes impaired during aging and this phenomenon is manifested earlier in 3xTg-AD mice. The increased incidence of astrocytes with limited ability to take up ions/neurotransmitters may further add to a cytotoxic environment.

## Introduction

Alzheimer’s disease (AD) is a chronic progressive neurodegenerative disorder which affects 35.6 million people worldwide, and the numbers are expected to double every twenty years (Prince et al., 2013). The neurodegeneration occurs in the prefrontal cortex, entorhinal cortex and hippocampus, which are brain structures related to learning and memory, and which are further linked to the limbic system associated with cognitive and emotional processes (Minati et al., 2009; Kulijewicz-Nawrot et al., 2012). Therefore, AD mainly affects the memory and cognitive functions and, in its later stage, results in dementia. Although the disease has been intensively studied for more than a hundred years and several processes underlying neuronal loss have been proposed during this period, the overall understanding of the AD pathology remains incomplete. The most frequently declared causes of AD comprise neurodegeneration driven by amyloid-β (Aβ) toxicity and tau pathology, synaptic dysfunction as well as the loss of synaptic density, glutamate excitotoxicity, and oxidative stress (Smith et al., 2000; Hardy and Selkoe, 2002; Ingelsson et al., 2004; Walton and Dodd, 2007; Kriska et al., 2021). However, the pathological processes behind AD not only cause neuronal death, but also involve functional changes in other cell types, such as glia or pericytes, alterations in inter-cellular communication, changes in the composition of perineural networks, and vascular system disruption (De Strooper and Karran, 2016; Goetzl and Miller, 2017).

Astrocytes represent multifunctional cells, which play an essential role in brain development, metabolism, in the control of the central nervous system (CNS) microenvironment, in modulating synaptic transmission and neurotransmitter release, and they also perform an important vascular function. During the progression of AD, astrocytes undergo morphological and functional changes and, ultimately, they become reactive (Kulijewicz-Nawrot et al., 2012, 2013; Acosta et al., 2017; Assefa et al., 2018). Impaired astrocytic functioning leads to altered glutamate (Glu) and ion homeostasis, as well as an increased release of Glu, γ-aminobutyric acid (GABA), cytokines and inflammatory mediators (Hynd et al., 2004; Ben Haim et al., 2015). Moreover, reactive astrocytes together with microglia, associate with Aβ deposits and are involved in neuritic plaque formation (Itagaki et al., 1989; Serrano-Pozo et al., 2013).

Here we focus on the AD related changes in the astrocytic uptake of ions and neurotransmitters from the extracellular space (ECS), which maintains their optimal concentration for the effective functioning of synaptic transmission. The intracellular accumulation of ions is followed by the entry of water, which leads to a transient increase in cell volume. In a healthy adult brain, astrocyte swelling triggers compensatory mechanisms that lead to an alleged regulatory volume decrease (RVD) (Okada et al., 2009; Wilson and Mongin, 2018). However, the ability of astrocytes to swell, as well as to regulate their volume, declines in the healthy aged brain. Under pathological conditions, in which the concentration of ions in the ECS increases significantly (Pasantes-Morales and Vazquez-Juarez, 2012), the compromised uptake of ions/neurotransmitters in astrocytes and/or loss of the ability to regulate their volume observed during aging may significantly add to the progression of AD. Such changes in astrocyte volume and morphology, together with the composition and structure of the extracellular matrix (ECM), affect the diffusion parameters of ECS. As the composition of ECS is crucial in the process of waste clearance, it is also feasible that changes in the ECS diffusivity contribute to the pathology of AD, due to the insufficient Aβ removal (Nedergaard and Goldman, 2020). Moreover, recent studies have shown that some ECM molecules can actively participate in the pathogenesis of the AD development, and promote an intracellular accumulation of protein aggregates such as Aβ, tau and α-synuclein (Holmes et al., 2013).

The main goal of this study was to assess astrocyte ability to control the ion or neurotransmitter uptake in the triple transgenic model of AD (3xTg-AD) containing three mutations associated with familial AD. To achieve this objective, we employed three-dimensional (3D) confocal morphometry, a method which enables the study of single-cell morphology changes during exposure to pathological stimuli, namely hypo-osmotic stress, or high K^+^ concentrations (Chvatal et al., 2007a,b). Cell volume changes mirror alterations in the expression/function of the different ion channels and transporters responsible for the uptake of ions and neurotransmitters, as well as for RVD. To identify such changes in the expression of astrocytic transport proteins, we employed a single-cell reverse transcription quantitative polymerase chain reaction (sc RT-qPCR). Finally, these observations were correlated with alterations in the diffusion parameters of ECS, reflecting both changes in cellular volume and ECS structure. All studied parameters were monitored during physiological aging and during the progression of AD.

## Materials and Methods

### Animals

Experimental animals were generated by crossbreeding of the triple transgenic model of AD, containing three mutations associated with familial AD (APP Swedish, MAPT P301L, and PSEN1 M146V) (Oddo et al., 2003) and GFAP/EGFP strain (Nolte et al., 2001). These hybrids enable the visualization of astrocytes for the use of morphological studies due to the enhanced green fluorescent protein (EGFP), expressed under the control of the human glial fibrillary acidic protein (GFAP) promoter. The experiments were performed on GFAP/EGFP mice (FVB/NJ, controls) and GFAP/EGFP x 3xTg-AD mice (C57Bl/6; hereinafter referred to as 3xTg-AD) at the ages of 3, 9, 12, and 18 months (3M, 9M, 12M, 18M).

All procedures involving the use of laboratory animals were performed in accordance with the European Communities Council Directive November 24, 1986 (86/609/EEC) and animal care guidelines approved by the Institute of Experimental Medicine ASCR Animal Care Committee, approval number 95/2015, 49/2019. All efforts were made to minimize both the suffering and the number of animals used. The mice were kept on a 12-h light/dark cycle with access to food and water *ad libitum*.

### Solutions

The compositions of brain isolation solution and artificial cerebrospinal fluid (aCSF), solution modeling hypo-osmotic stress (aCSF_H–100_), and solution modeling hyperkalemia (aCSF_K+_) are listed in Table 1. All solutions were equilibrated with 95% O_2_ and 5% CO_2_ (Carbogen, Siad, Branany, Czechia) to a final pH of 7.4, and osmolality of ∼300 mOsmol/kg was measured using a vapor pressure osmometer (Vapro 5520, Wescor, Logan, UT, United States).

**TABLE 1:** Composition of the solutions.

Compounds	Isolation solution (mM)	aCSF (mM)	aCSF_H–100_ (mM)	aCSF_K+_ (mM)
NaCl	-	122	67	75
NMDG	110	-	-	-
KCl	2.5	3	3	50
NaHCO_3_	24.5	28	28	28
NaHPO_4_	1.25	1.25	1.25	1.25
Glucose	20	10	10	10
CaCl_2_	0.5	1.5	1.5	1.5
MgCl_2_	7	1.3	1.3	1.3
Osmolality (mOsmol/kg)	300 ± 10	300 ± 10	200 ± 10	300 ± 10

*Key: NMDG, N-methyl-D-glucamine; aCSF, artificial cerebrospinal fluid; aCSF_H–100_, hypo-osmotic solution; aCSF_K+_, solution with elevated potassium concentration.*

### Preparation of Acute Brain Slices

The experimental mice were deeply anesthetized with 1% pentobarbital (100mg/kg; i.p.), transcardially perfused with cold (4 – 8°C) isolation solution (Table 1), and decapitated. The brains were dissected out and coronal 300-μm-thick slices were cut using a vibrating microtome (Leica Biosystems, Buffalo Grove, IL, United States). The slices were incubated for 40 min at 34°C in the isolation solution, and then transferred to aCSF (Table 1) in which they were kept throughout the experiment at room temperature. To minimize the damage to the nervous tissue isolated from aged mice, N-methyl-D-glucamine-based isolation solution (Table 1) was always used during slice preparation and incubation after the brain cutting, as described for aged mice/rats in the study by Ting et al. (2014).

### Real-Time Iontophoretic Method

The real-time iontophoretic (RTI) method was used to define the ECS diffusion parameters: ECS volume fraction (α), tortuosity (λ), and nonspecific uptake (k’) (Nicholson and Phillips, 1981; Sykova and Nicholson, 2008). ECS volume fraction is the space available for diffusion and is defined as the ratio of the volume of the ECS to total tissue volume in a representative volume of brain tissue (α = ECS/total tissue volume). Tortuosity is defined as square root of the ratio of the free diffusion coefficient in a medium with free diffusion, and the apparent diffusion coefficient in the brain tissue. Tortuosity reflects the compromised extracellular diffusion in the tissue in comparison with a free diffusion medium due to the presence of diffusion barriers created, for example, by cell processes, ECM molecules, or “dead spaces” (Hrabetova et al., 2003; Thorne and Nicholson, 2006; Sykova and Nicholson, 2008). Nonspecific uptake represents the loss of diffusing ions/molecules from the ECS into cells or capillaries.

In brief, an extracellular marker, tetramethylammonium ions (TMA^+^, MW = 74.1), to which cell membranes are relatively impermeable, are introduced into the tissue through the iontophoretic microelectrode, and imitate the extracellular diffusion of small ions and molecules. The time-dependent changes in the concentration of TMA^+^ in the ECS are determined by ion-selective microelectrodes (ISMs). Double-barrelled TMA^+^-ISMs were prepared so that the tip of the ion-sensitive barrel was filled with an ion-exchanger IE190 (WPI, Inc., Sarasota, FL, United States, RRID:SCR_008593); the rest of the barrel was backfilled with 100 mM TMA^+^. The reference channel contained 150 mM NaCl. Prior to the experiments, TMA^+^-ISMs micropipettes were calibrated using the fixed-interference method in a series of five different concentrations of TMA^+^ (mM): 0.1, 0.3, 1.0, 3.0, 10.0 in a background of 150 mM NaCl and 3 mM KCl. The signal was recorded by a locally constructed differential amplifier (Voipio et al., 1994), which subtracts the signals from the reference and ion-selective barrels. Calibration voltages were fitted to the Nikolsky equation, to determine the slope and the interference of each ISM (Nicholson, 1993).

The TMA^+^-ISM and an iontophoretic micropipette, backfilled with 100 mM TMA^+^ chloride, were glued together using dental cement with a tip separation of 90–150 μm. The 20 nA bias current was applied continuously (Single Channel Iontophoresis Generator ION-100, Dagan Corporation, Minneapolis, MN, United States) to maintain a constant electrode transport number. The diffusion curves were generated by a current step to 200 nA of 24 s duration (Master 8, A.M.P.I, Jerusalem, Israel) and captured on a digital oscilloscope (NIC Nicolet Dual Channel Digital Oscilloscope 3091). In order to calibrate the electrode array before tissue measurements, the diffusion curves were recorded in 0.3% agar gel (Sigma Aldrich, Germany), dissolved in a solution of 150 mM NaCl, 3 mM KCl and 1 mM TMA^+^. The curves were then analyzed by a non-linear curve fitting simplex algorithm, operating on a modified diffusion equation, using the VOLTORO program (kindly provided by C. Nicholson, New York University School of Medicine, United States). Calibration determines the values of the free diffusion coefficient of TMA^+^ (D), the distance between electrodes (r) and the transport number (n_t_), a dimensionless parameter that denotes the fraction of the applied current that carries the TMA^+^ out of the micropipette during an iontophoretic pulse. With the known D, r and n_t_-values, the parameters α (volume fraction), λ (tortuosity) and k’ (non-specific uptake) can be determined.

Data are presented as the mean ± SEM. Statistical analyses for diffusion measurements were performed by Student’s *t*-test with Benjamini-Hochberg *post hoc* test to control the false discovery rate when conducting multiple comparisons (age-related differences within control or 3xTg-AD mice) or two-way ANOVA test with Tukey *post hoc* test (differences between control and age-matched 3xTg-AD mice). Differences between the groups were considered statistically significant when *p* < 0.05, very significant when *p* < 0.01, and extremely significant when *p* < 0.001.

### Cell Volume Measurement and Quantification

Time-dependent changes in astrocyte volume were studied using 3D - confocal morphometry in the CA1 region of hippocampal slices. The method was previously described by Chvatal et al. (2007a,b). During all the experiments, brain slices were placed into the recording chamber and fixed with a U-shaped platinum wire with a grid of nylon threads. The recording chamber was continuously perfused with aCSF or solutions modeling hypo-osmotic stress (aCSF_H–100_) or hyperkalemia (aCSF_K+_). Cell images for the volume quantification were taken every 5 min during the 20-min exposure to aCSF_H–100_ or aCSF_K+_ and every ten minutes during the following 40-min washout. The cells were recorded as a set of two-dimensional (2D) sectional images, with a resolution of 1024 × 1024 pixels using FV1200MPE confocal microscope with 60 × LUMPLFLN water objective (Olympus, Shinjuku, Japan). EGFP was excited by an Ar laser at 488 nm, and the emitted signal was recorded over the range of 488 nm using a DM405/488 filter. Each 3D image of the cell was sectioned into 65 - 85 consecutive 2D images at a uniform spacing of 1 μm. To avoid the inclusion of damaged cells into the experiments, especially those with partly damaged processes in the vicinity of the slice surface, we always chose cells with processes emerging 20 – 30 μm beneath the surface were always chosen, as described earlier in our previous work (Benesova et al., 2009).

Image processing and morphometric measurements were performed using the program Cell Analyst, developed at the Department of Cellular Neurophysiology, Institute of Experimental Medicine, Prague, Czechia (Chvatal et al., 2007a,b). For each time point, 4 - 5 mice were used, and 2 - 3 slices were prepared from the hippocampus of both hemispheres. These half-slices were used for quantification of the volume of 1 or 2 EGFP-positive cells. Changes in the total astrocyte volume are presented as the mean ± SEM. Statistical significance was determined in the time course of astrocyte volume changes during aging by two-way ANOVA with Tukey’s *post hoc* test. Differences between the groups were considered statistically significant when *p* < 0.05, very significant when *p* < 0.01, and extremely significant when *p* < 0.001.

### Immunohistochemistry

The mice were deeply anesthetized with pentobarbital (100 mg/kg, i.p.), and transcardially perfused with 20 ml of saline with heparin (2500 IU/100 mL; Zentiva, Prague, Czechia) followed by 20 ml of 4% paraformaldehyde. The brains were dissected, postfixed in 4% paraformaldehyde overnight, and placed stepwise in solutions with gradually increasing sucrose concentrations (10, 20, and 30%) for cryoprotection. Coronal slices (30 μm) were prepared using Hyrax C50 cryostat (Zeiss, Gottingen, Germany). The slices were incubated in a blocking solution containing 5% ChemiBLOCKER (Merck, Darmstadt, Germany) and 0.5% Triton X-100 (Merck, Darmstadt, Germany) in phosphate buffer saline (PBS) for 1 h. They were then incubated overnight at 4°C with primary antibodies diluted in a blocking solution, followed by a 2-h incubation with species-specific secondary antibodies diluted in blocking solution at room temperature. Primary antibody against Amyloid beta (1:1000, EMD Millipore; Merck, Darmstadt, Germany) was used. Corresponding secondary antibody (goat anti-rabbit IgG, goat anti-mouse IgG, conjugated with Alexa-Fluor 594; Thermo Fisher Scientific; Waltham, MA, United States) was diluted to 1:200. To visualize the cell nuclei, the slices were incubated with 300 nM 4′, 6-diamidino-2-phenylindole (DAPI) in PBS for 5 min at room temperature. After immunostaining, the slices were mounted onto microscope slides using Aqua-Poly/Mount (Polysciences Inc, Eppelheim, Germany).

### Gene Expression Profiling

#### Preparation of Single Cell Suspension From the Hippocampal CA1 Region

The mice aged 3, 9, 12, and 18 M were anesthetized with sodium-pentobarbital (100 mg/kg, i.p.), and perfused transcardially with cold (4 – 8 °C) isolation buffer containing (in mM) NaCl 136.0, KCl 5.4, Hepes 10.0, glucose 5.5, osmolality 290 ± 3 mOsmol/kg. The hippocampus was removed and used for the preparation of cell suspension using a papain dissociation kit (Worthington, Lakewood, NJ, United States). The dissociated cells were layered on top of 5 ml of Ovomucoid inhibitor solution (Worthington, Lakewood, NJ, United States) and subsequently harvested by centrifugation (140 × *g* for 6 min). This method routinely yielded ∼2 × 10^6^ cells per mouse brain. Cell aggregates were removed by filtering with 70 μm cell strainers (Becton Dickinson, NJ, United States), and the cells were kept on ice until sorting.

#### Collection of Single Enhanced Green Fluorescent Protein-Positive Cells

The astrocytes were collected using flow cytometry (fluorescence-activated cell sorting [FACS]; BD Influx, San Jose, CA, United States). FACS was calibrated manually to deposit single cells in the center of each collection tube. Hoechst 33258 (Life Technologies, Carlsbad, CA, United States) was added to the suspension of cells to check cell viability. Single cells were sorted into 96-well plates (Life Technologies), each well contained 5 mL of nuclease-free water with bovine serum albumin (1 mg/ml, Fermentas, Rockford, IL, United States) and RNaseOut 20 U (Life Technologies). The plates were then placed on a precooled rack. The astrocytes were collected based on their positivity for EGFP and their viability. The plates with collected cells were immediately frozen at 80°C and stored until the analysis.

#### Single-Cell Gene Expression Profiling

The collected single cells were analyzed by sc RT-qPCR. In total, the expression of 96 genes was determined (Supplementary Table 1). Primers were designed using PrimerBLAST. When possible, each primer pair was separated by at least one intron on the corresponding genomic DNA. For each assay, specificity was tested by melt curve analysis and gel electrophoresis. The effectivity of each assay was determined using a standard dilution over 6 orders of magnitude. In RT-qPCR analysis, samples were reverse-transcribed into cDNA using SuperScript III (ThermoFisher Scientific). The reverse transcription was performed using the standard protocol recommended by the manufacturer, except with the total volume of 10 μl, equimolar mix of oligo-dT and random hexamers (50 μM) and a reduced concentration of SuperScript III enzyme (50 U), was used. To monitor the risk of inhibition, RNA TATAA Universal RNA Spike II was added into each reaction based on the manufacturer’s instructions (TATAA Biocenter, Sweden). Five microliters of non-diluted cDNA were further pre-amplified using a mix of all primers. Preamplified cDNA was diluted 4 times and analyzed using BioMark instrument (Fluidigm, United States). A detailed description of each procedure can be found elsewhere (Rusnakova et al., 2013; Valny et al., 2018).

#### Data Processing

In total, 461 cells from 37 mice were analyzed (3M: 4 controls, 4 3xTg-AD, 9M: 4 controls, 5 3xTg-AD, 12M: 6 controls, 5 3xTg-AD, 18M: 4 controls, 5 3xTg-AD). RT-qPCR data were pre-processed in Fluidigm Real-Time PCR Analysis software (4.1.2, Fluidigm, United States) and analyzed with GenEx software (Ver. 6.1.1.550, MultiD, Sweden). *C*_q_-values measured from amplifications that generated melting curves with aberrant T_m_ were removed, as well as *C*_q_-values larger than 28. All missing data, for each gene separately, were then replaced with the highest *C*_q_ +2 (25% of the lowest measurable concentration). *C*_q_-values with non-missing data were transformed into relative quantities (scaled to the sample with the lowest expression) and converted into log 2 scale. The data are presented as the mean ± SEM. Statistical analyses of the differences in gene expression among groups were performed using ANOVA for multiple comparisons, with Tukey’s *post hoc* test, or Student’s *t*-test when appropriate. Differences between the groups were considered statistically significant when *p* < 0.05, very significant when *p* < 0.01, and extremely significant when *p* < 0.001.

## Results

We hypothesized that morphological changes of individual cellular elements or changes in the composition of the ECM during AD, may affect the volume and diffusion properties of ECS. To test this hypothesis, we performed measurements of the extracellular diffusion parameters in the CA1 region of hippocampal slices by the RTI method during the exposure to the pathological models of hypo-osmotic stress and hyperkalemia. Additionally, the astrocyte functional changes, such as the uptake of ions from ECS and the ability to regulate their volume, were tested using the 3D confocal morphometry method. Finally, we used sc RT-qPCR to assess expression changes of genes which are responsible for astrocytic key homeostatic functions. All experiments were performed in 3-, 9-, 12- and 18M controls and 3xTg-AD mice. Since no sex differences were noted, data from males and females were pooled.

### Validation of the Triple Transgenic Mouse Model of Alzheimer’s Disease

**FIGURE 1:**
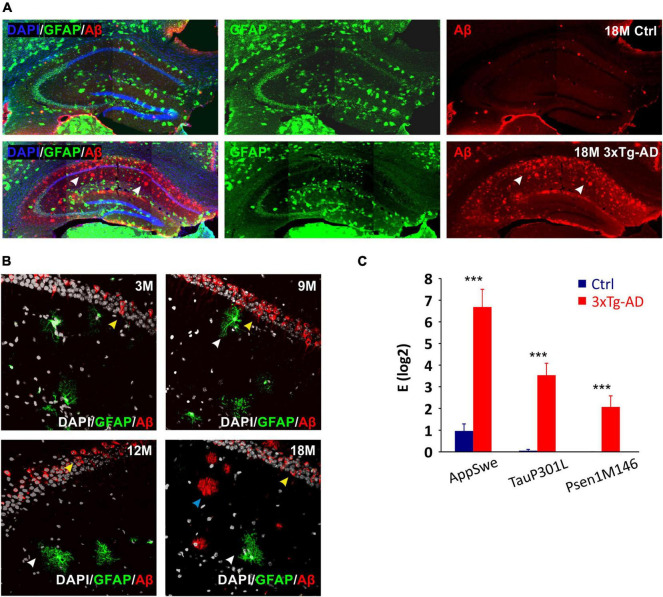
Confirmation of the 3xTg-AD phenotype in hybrids generated by crossbreeding with GFAP/EGFP mice. **(A)** Overview images of amyloid-β (Aβ) expression in hippocampus of 18M-old controls and 3xTg-AD mice. White arrows point to the expression of amyloid plaques in the CA1 region of the hippocampus in a transgenic model of Alzheimer’s disease. **(B)** Details of Aβ expression in hippocampal CA1 area of 3-, 9-, 12- and 18M-old mice. Yellow arrows point to the intracellular expression in pyramidal neurons (all ages), blue arrows point to the extracellular plaques (18 months only) and white arrows point to the EGFP labeled astrocytes lacking intracellular Aβ expression. **(C)** Relative mRNA expression of 3xTg-AD marker genes in EGFP-positive hippocampal astrocytes isolated from 3M-old mice. The expression of all three mutated genes was maintained in hybrid mice. Data are presented as mean ± SEM. Statistical significance was determined by Student’s *t*-test. Asterisks indicate significant differences (***p < 0.001). Ctrl, control; 3xTg-AD, triple transgenic model of AD crossbred with GFAP/EGFP mice; GFAP, glial fibrillary acidic protein; EGFP, enhanced green fluorescent protein; Aβ, amyloid-β; AppSwe, Swedish mutation in amyloid precursor protein gene; TauP301L, P301L mutation of tau protein gene; Psen1M146, M146V mutation of presenilin1 gene; 3M, 9M, 12M, 18M, 3-, 9-, 12-, 18-month-old animals.

For the purpose of the morphological study, the 3xTg-AD mice (Oddo et al., 2003) were crossbred with GFAP/EGFP mice (Nolte et al., 2001). The hybrid mice retained the 3xTg-AD phenotype and in addition, they expressed EGFP under the GFAP promoter, allowing the visualization of astrocytes. Immunohistochemical staining of Aβ confirmed its expression in the motor cortex and hippocampus, similarly to the original model (Figure 1A). In the hippocampal CA1 region, the intracellular expression in pyramidal neurons was already observed in the 3M mice, while the external presence of Aβ plaques was not evident until 18 months of age (Figure 1B). Single-cell RT-qPCR verified the expression of all three mutations (AppSwe, TauP301L, Psen1M146) in EGFP-positive cells (Figure 1C).

### Diffusion Parameters in the Hippocampus of Triple Transgenic Mouse Model of Alzheimer’s Disease

**FIGURE 2:**
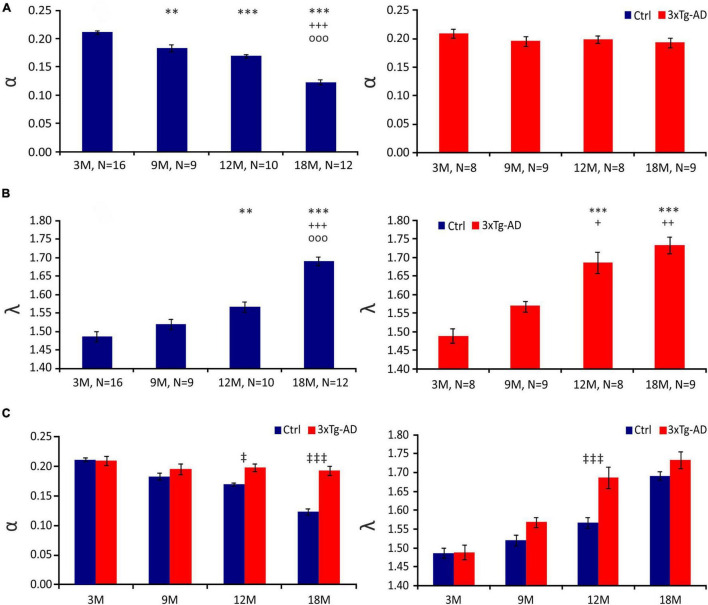
Diffusion parameters of extracellular space during aging of control (Ctrl) and 3xTg-AD mice at 3, 9, 12 and 18 months (3, 9, 12 and 18M). Average values of the volume fraction α **(A)** and tortuosity λ **(B)** during aging in Ctrl (left) and 3xTg-AD mice (right). Note the continuous decrease in α during a physiological aging which is missing in 3xTg mice. A marked increase in λ occurred in 3xTg-AD animals earlier than in Ctrl mice. Comparison of values of α (left) and λ (right) between Ctrl and age-matched 3xTg-AD mice **(C)**. Data are expressed as mean ± SEM. Statistical significance was determined by Student’s *t*-test with Benjamini-Hochberg post-hoc test. Asterisks, crosses, circles and double daggers indicate significant differences (*p* < 0.05 [*/+/o/‡ significant]; *p* < 0.01[^**^/++/oo/‡‡ very significant]; p < 0.001 [^***^/+++/ooo/‡‡‡ extremely significant]). * indicates a significant difference related to the values of 3M-old mice; + indicates a significant difference related to the values of 9M-old mice; o indicates a significant difference related to the values of 12M-old mice, ‡ indicates a significant difference between Ctrl and age-matched 3xTg-AD mice. Ctrl, control mice; 3xTg-AD, triple transgenic model of AD; 3M, 9M, 12M, 18M, 3-, 9-, 12-, 18-month-old animals; N, number of animals.

In this study, we identified a significant continuous decrease in α during physiological aging in the CA1 region of the hippocampus (3M - ∼0.212; 9M - ∼0.188; 12M - ∼0.169; 18M - ∼0.123). In contrast, we detected no age-related decrease in α in 3xTg-AD mice (3M - ∼0.212; 9M - ∼0.188; 12M - ∼0.199; 18M - ∼0.197) (Figure 2A). The values of α in 3xTg-AD mice and their age-matched controls began to differ significantly at 12 months of age; this difference was extremely significant at 18 months (Figure 2C).

In the CA1 region of the hippocampus of the control mice, we observed an increasing tendency in the λ values, which were significantly higher in 12M and 18M mice than in 3M mice. In 18M mice, an additional increase in λ was detected; the value was significantly higher in comparison with all other age groups (3M - ∼1.491; 9M - ∼1.543; 12M - ∼1.560 ± 0.017; 18M - ∼1.695 ± 0.016). The data of the 3xTg-AD mice also showed that λ increases over age (Figure 2B). This increase was faster than in the control animals, and most pronounced in 12M and 18M mice (3M - ∼1.511; 9M - ∼1.562; 12M - ∼1.650; 18M - ∼1.736). The values of λ in 12M mice were significantly higher in the 3xTg-AD mice than in the age-matched controls, but the difference disappeared in 18M mice (Figure 2C).

In conclusion, physiological aging was accompanied by a decrease in ECS volume fraction, while this tendency was completely lost during AD progression. However, an increase in the number of obstacles observed in AD progression occurred earlier than in physiological aging.

### Changes in the Diffusion Parameters of Extracellular Space Induced by Hypo-Osmotic Stress or Hyperkalemia

To further disclose the differences between 3xTg-AD mice and controls, experimental cell swelling in brain slices was evoked by exposure to hypo-osmotic stress (aCSF_H–100_) or severe hyperkalemia (aCSF_K+_). The ECS diffusion parameters were determined during basal conditions and during a 20-min perfusion with aCSF_H–100_ or aCSF_K+_, followed by a 40-min application of aCSF (further termed washout). As the basal values of α and λ varied, we evaluated both the absolute and relative changes of the ECS parameters. To estimate the relative changes, the control values were set to 100%, and the relative changes were calculated.

**FIGURE 3:**
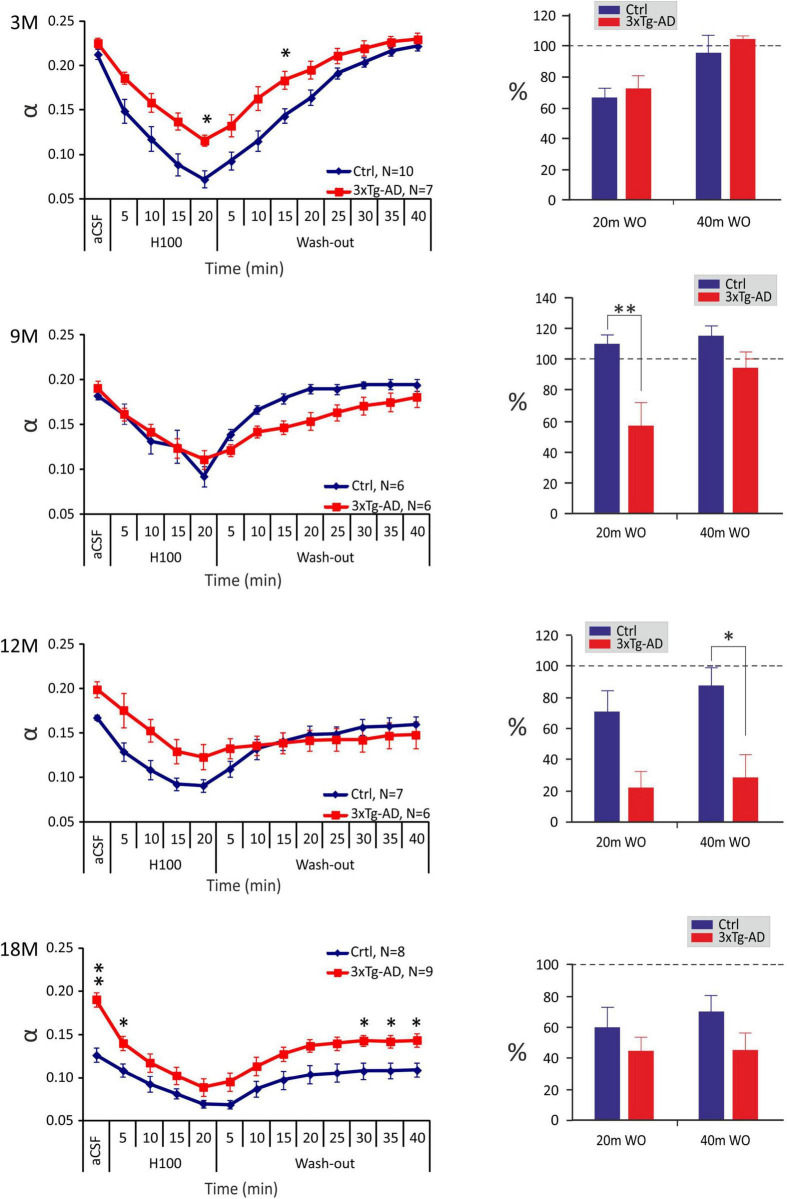
Age-dependent changes of the absolute values of the ECS diffusion parameters in control and 3xTg-AD mice evoked by hypo-osmotic stress. The left side: Time course of the volume fraction (α) changes during a 20-min application of aCSF_H–100_ and a 40-min washout in 3-, 9-, 12- and 18M-old control animals and age-matched 3xTg-AD mice. The right side: Volume fraction recovery during washout at 20 min intervals is expressed as changes in the values reached in the 20th min of application, set as 0%. Data are presented as mean ± SEM. Statistical significance was determined by two-way ANOVA test with Tukey’s *post hoc* test. Asterisks indicate significant differences between Ctrl and 3xTg-AD mice (*p < 0.05, ^**^p < 0.01). Ctrl, control mice; 3xTg-AD, triple transgenic model of AD; aCSF, artificial cerebrospinal fluid; H100, hypotonic artificial cerebrospinal fluid; WO, wash-out; 3M, 9M, 12M, 18M, 3-, 9-, 12-, 18-month-old animals; N, number of animals.

In all ages and groups, the application of aCSF_H–100_ induced a significant decrease in α with a complete or partial recovery during washout (Figure 3). A significant difference between the control and 3xTg-AD mice in the minimal α values during the application (20 min after the induction of hypo-osmotic stress), was observed only at 3 months of age, where the values of α were lower in the control mice (∼0.072) than in 3xTg-AD mice (∼0.116). Despite the higher basal α values in 3xTg-AD mice in 18M mice, the minimal values at 20 minutes of application did not differ between the control and 3xTg-AD mice. During the washout in the control animals, we detected a complete recovery of α in age of 3-, 9-, and 12 months, and partial recovery in the oldest, 18M group. In 3xTg-AD mice, α fully recovered to basal values in 3M and 9M animals; only partial recovery was detected in 12- and 18M- old mice. To further analyze the ECS volume changes during the washout period, they were expressed as a percentage of volume increase/decrease in relation to the value of α reached in the 20th min of application, which was set as 0%. This analysis showed a significant difference between the control and 3xTg-AD animals in the 20th min of washout in 9M mice and the 40*^th^* minute of washout in 12M mice (Figure 3). These results already indicate a slower and compromised recovery in the 3xTg-AD mice in 9M- and 12M-age groups. Tortuosity increased during the application of aCSF_H–100_ and returned to the basal values during washout in all the groups. The only difference between the control and 3xTg-AD mice was observed at 12M, where λ values were higher in the 3xTg-AD mice than in controls during almost the entire experiment due to a distinctly larger basal value (Supplementary Figure 1A). Relative values of volume fraction and tortuosity did not show any significant changes between the control and 3xTg-AD mice (Supplementary Figure 2).

In summary, changes in ECS volume fraction and tortuosity evoked by hypo-osmotic stress were comparable between the 3xTg-AD mice and their age-matched controls, however compromised and slower recovery of ECS volume fraction in the 3xTg-AD mice was detected in 9- and 12M mice.

**FIGURE 4:**
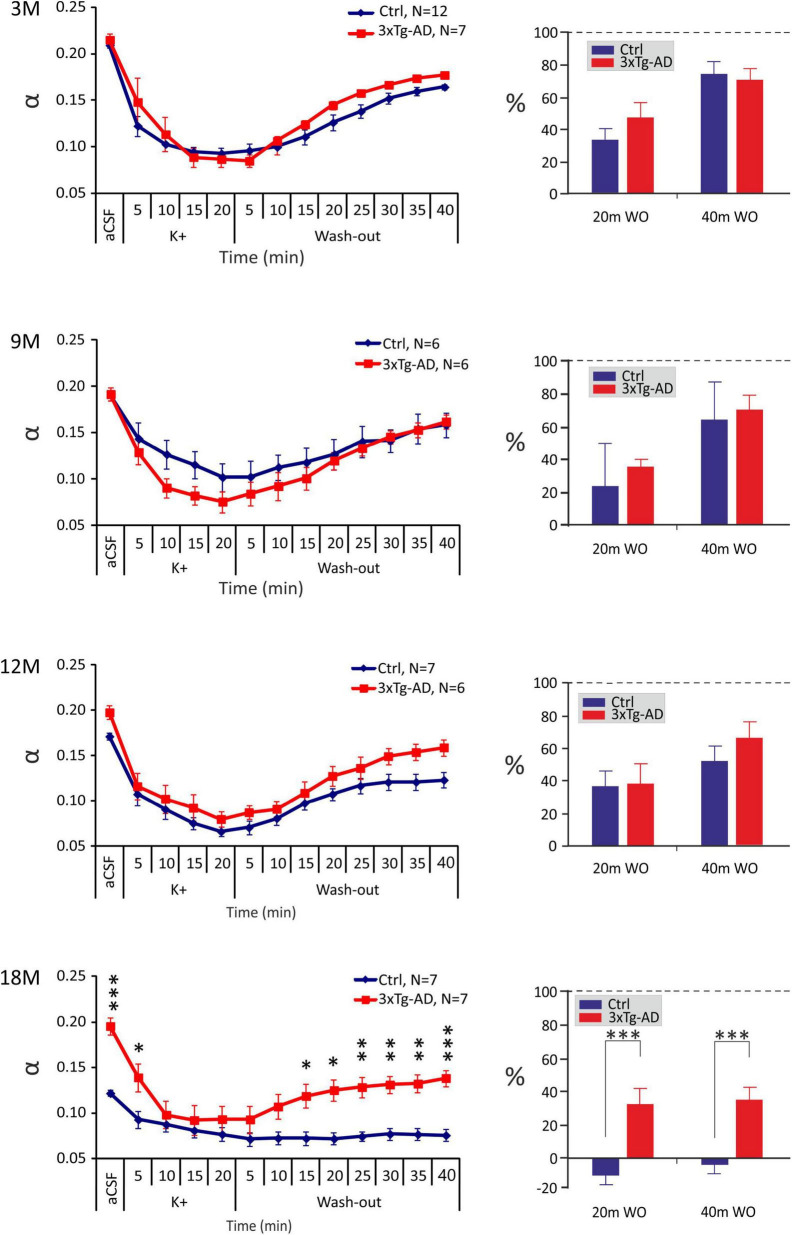
Age-dependent changes of the absolute values of the ECS diffusion parameters in control (Crtl) and 3xTg-AD mice evoked by severe hyperkalemia (50 mM K^+^). The left side: Time course of the volume fraction (α) changes during a 20-min application of aCSF_K+_ and a 40-min washout in 3-, 9-, 12- and 18M-old control animals and age-matched 3xTg-AD mice. The right side: Volume fraction recovery during washout at 20 min intervals is expressed as changes in the values reached in the 20th min of application, set as 0%. Data are presented as mean ± SEM. Statistical significance was determined by two-way ANOVA test with Tukey’s *post hoc* test. Asterisks indicate significant differences between Ctrl and 3xTg-AD mice (*p < 0.05, ^**^p < 0.01, ^***^p < 0.001). Ctrl, control mice; 3xTg-AD, triple transgenic model of AD; aCSF, artificial cerebrospinal fluid, K+, artificial cerebrospinal fluid with elevated K+ concentration; WO, wash-out; 3M, 9M, 12M, 18M, 3-, 9-, 12-, 18-month-old animals; N, number of animals.

The application of the aCSF_K+_ solution evoked a significant decrease of α in all the experimental groups, followed by a partial recovery towards basal values during washout with aCSF solution, apart from 18M control mice, where no recovery was detected (Figure 4). Significant differences between the control and 3xTg-AD mice were only observed at 18M of age, where α values in the 3xTg-AD animals where higher than those in the controls before the application and during washout but the minimal values reached during the application did not differ. The recovery analysis showed a significant difference between the control and 3xTg-AD animals in the 20th and 40th min of washout in 18M-old mice (Figure 4). While the values of α in the oldest control group did not recover at all, partial recovery was still detected in the 3xTg-AD mice, presumably due to the higher initial value of α. Severe hyperkalemia evoked an increase in λ in all experimental groups, followed by a recovery to basal values during washout. Similarly, to aCSF_H–100_ application, we only found differences between the control and 3xTg-AD mice at 12 months of age, where λ values in the 3xTg-AD mice during the basal conditions, aCSF_K+_ application and washout were higher than those in the controls (Supplementary Figure 1B). The relative values of volume fraction and tortuosity did not show any significant differences between the control and 3xTg-AD mice (Supplementary Figure 3). The complete data are summarized in Supplementary Tables 2, 3.

In summary, comparable changes in ECS volume fraction and tortuosity evoked by hyperkalemia, were observed between the 3xTg-AD mice and their age-matched controls within 3 – 12M of age. However, in 18M mice, due to a higher initial ECS volume fraction a better recovery in the 3xTg-AD than in control mice was detected.

### Astrocyte Swelling Induced by Hypo-Osmotic Stress or High Extracellular K^+^ Concentration in Triple Transgenic Mouse Model of Alzheimer’s Disease

Swelling of astrocytes and their ability of volume regulation were tested in acute brain slices from 3M, 9M, 12M, and 18M mice exposed to aCSF_H–100_ or aCSF_K+_. The 20-min exposure was followed by a 40-min washout. Changes in the total astrocytic volume (ΔV_c_) were recorded every 5 min during the aCSF_H–100_ or aCSF_K+_ application and every 20 min during the washout. The astrocyte volume at *t* = 0 was set to 100% and the astrocytic swelling was expressed relative to this baseline as a percentual increase/decrease.

**FIGURE 5:**
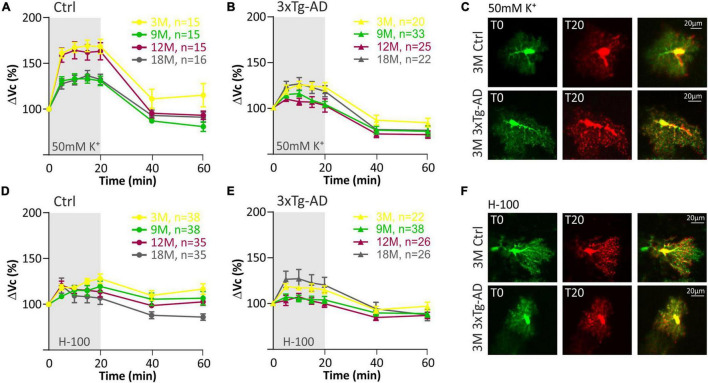
Swelling of hippocampal astrocytes in response to severe hyperkalemia (50 mM K^+^) or hypo-osmotic stress in aging controls and 3xTg-AD mice. **(A,B)** Time course of the total volume changes (ΔVc) in EGFP-positive cells during a 20-min application of aCSF_K+_ and a 40-min washout. **Note** that in controls (left) the astrocyte swelling is comparable in 3M- and 12M- and in 9M- and 18M-old animals. No such age-related changes were observed in 3xTg-AD mice (right). **(C)** Representative images of EGFP-expressing astrocytes before (T0, red) and after the 20-minute exposure to 50 mM K^+^ (T20, green). Overlays show merged images of cells with their initial volume (red) and swollen cells (green). Green area represents swelling induced by 50 mM K^+^. **(D,E)** Time course of the total volume changes (ΔVc) in EGFP-positive cells during a 20-min application of aCSF_H–100_ and a 40-min washout. **Note** that no changes were observed between age groups in either controls or 3xTg-AD. **(F)** Representative images of EGFP-expressing astrocytes before (T0, red) and after the 20-min exposure to H-100 (T20, green). Overlays show merged images of cells with their initial volume (red) and swollen cells (green). Green area represents swelling induced by H-100. Data are presented as mean ± SEM. Statistical significance was determined by two-way ANOVA test with Tukey’s *post hoc* test. Ctrl, control; 3xTg-AD, triple transgenic model of AD crossbred with GFAP/EGFP mice; GFAP, glial fibrillary acidic protein; EGFP, enhanced green fluorescent protein; aCSF, artificial cerebrospinal fluid; H-100, hypotonic artificial cerebrospinal fluid; 50 mM K^+^, artificial cerebrospinal fluid with elevated K^+^ concentration; 3M, 9M, 12M, 18M, 3-, 9-, 12-, 18-month-old animals; n, number of cells.

In our previous study (Kolenicova et al., 2020) we showed that the volume of astrocytes induced by hyperkalemia varies with age. While in 3- and 12M animals astrocytes swelled markedly (up to a maximum of ∼170% in 3M), in 9- and 18M mice, the swelling was lower (maximum ∼140 % in 18M). These differences were not observed under the exposure to hypo-osmotic stress, mirroring that hyperkalemia activates different mechanisms responsible for the swelling than hypo-osmotic stress. In the present study, such age-dependent cell volume variations were not observed in astrocytes from 3xTg-AD mice in response to hyperkalemia as only minor differences were observed between the age groups (Figure 5; Supplementary Table 5). Similar to physiological aging, hypo-osmotic stress revealed no age-related differences in astrocyte swelling of 3xTg-AD mice (Figure 5; Supplementary Table 4).

**FIGURE 6:**
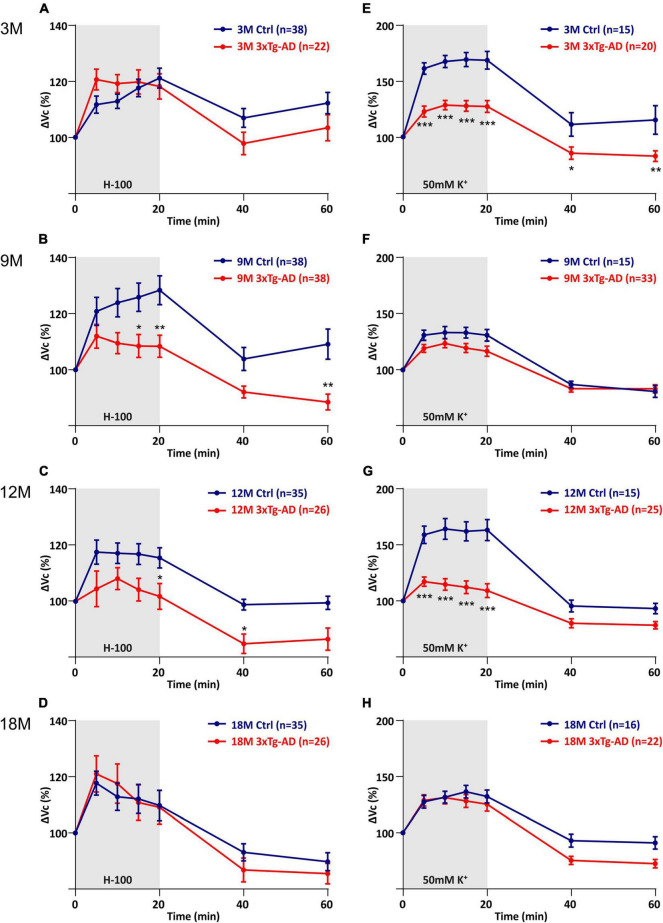
Comparison of astrocyte swelling in controls and 3xTg-AD during exposure to hypo-osmotic stress or severe hyperkalemia (50 mM K^+^). **(A–D)** Time course of the total volume changes (ΔVc) in EGFP-positive cells during a 20-min application of aCSF_H–100_ and a 40-min washout. **Note** that the astrocyte swelling is comparable in 3M- and 18M-old animals, while the astrocytic swelling in 9M- and 12M-old controls significantly exceeds the swelling in 3xTg-AD. **(E–H)** Time course of the total volume changes (ΔVc) in EGFP-positive cells during a 20-min application of aCSF_K+_ and a 40-min washout. **Note** that the astrocyte swelling is comparable in 9M- and 18M-old animals, while the astrocytic swelling in 3M- and 12M-old controls significantly exceeds the swelling in 3xTg-AD. Data are presented as mean ± SEM. Statistical significance was determined by two-way ANOVA with Tukey’s multiple comparison test. Asterisks indicate significant differences (*p < 0.05, **p < 0.01, ***p < 0.001). Ctrl, control; 3xTg-AD, triple transgenic model of AD crossbred with GFAP/EGFP mice; GFAP, glial fibrillary acidic protein, EGFP, enhanced green fluorescent protein; aCSF, artificial cerebrospinal fluid; H-100, hypotonic artificial cerebrospinal fluid; 50 mM K^+^, artificial cerebrospinal fluid with elevated K^+^ concentration; 3M, 9M, 12M, 18M, 3-, 9-, 12-, 18-month-old animals; n, number of cells.

The quantification of volume changes evoked by the hypo-osmotic stress in 3M and 18M animals revealed no differences between the controls and 3xTg-AD mice. In 3M mice ΔV_c_ reached the maximal values of ∼120% in the controls, and ∼121% in the 3xTg-AD (Figure 6A). In 18M mice it was ∼118% in the controls and ∼121% in 3xTg-AD mice (Figure 6D). Significant differences between the controls and 3xTg-AD mice were observed in both 9- and 12M. In 9M mice, the astrocyte volume increased to ∼128% in the controls, while it reached only ∼108% in 3xTg-AD after 20 minutes of aCSF_H–100_ application (Figure 6B). In 12M mice, the volume increased to the maximum values of ∼119% in the controls and ∼109% in 3xTg-AD (Figure 6C). The complete data are summarized in Supplementary Table 4.

The quantification of volume changes evoked by hyperkalemia in 9M and 18M animals revealed no differences between the controls and 3xTg-AD mice. In 9M mice, ΔV_c_ reached the maximal values of ∼133% in the controls and ∼123% in 3xTg-AD (Figure 6F). In 18M mice, it was ∼136.6% in the controls and ∼131% in 3xTg-AD mice (Figure 6H). Significant differences between control and 3xTg-AD were observed in 3M and 12M mice. In 3M mice, the astrocyte swelling reached maximal values of ∼169% in the controls, while only ∼129 in 3xTg-AD (Figure 6E). In 12M mice, the highest values of swelling reached to ∼164% in the controls and only ∼117% in 3xTg-AD (Figure 6G). The complete data are summarized in Supplementary Table 5.

Overall, we found that astrocyte swelling due to both hypo-osmotic stress and hyperkalemia was lower in the 3xTg-AD mice than in the controls. In addition, the astrocytic swelling in the AD model did not change with increasing age.

**FIGURE 7:**
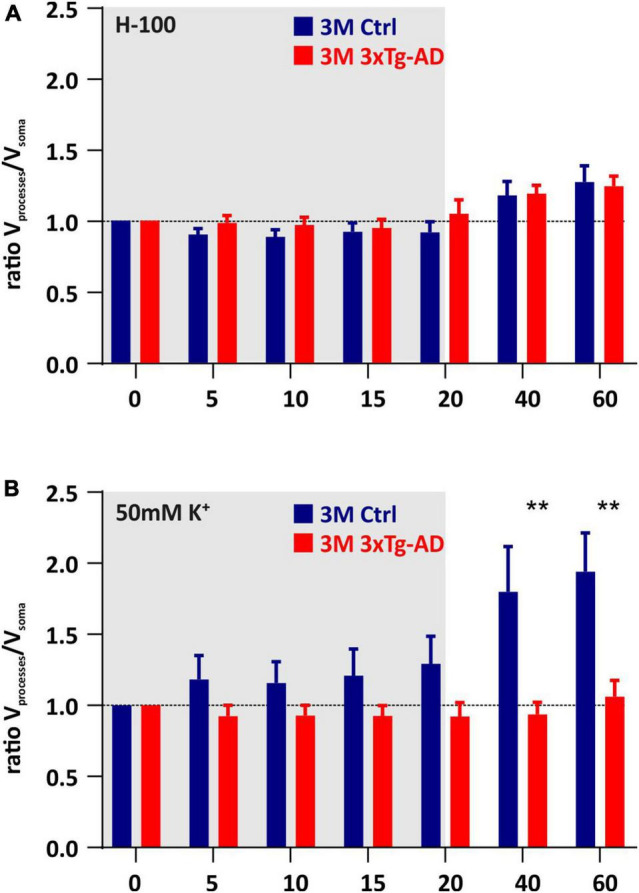
Contribution of cell somas/processes to the total volume changes induced by hypoosmotic stress **(A)** or hyperkalemia **(B)**. The bar graph shows the ratio of the change of processes volume to the change in soma. **Note** that under hyperkalemia, swelling of the processes predominates over the swelling of the somas, in the 3M-old controls, while in the respective age group in 3xTg-AD mice the somas and processes swell comparatively. Data are presented as mean ± SEM. Statistical significance was determined by two-way ANOVA test with Tukey’s *post hoc* test. Asterisks indicate significant differences (**p < 0.01). Ctrl, control; 3xTgAD, triple transgenic model of AD crossbred with GFAP/EGFP mice; GFAP, glial fibrillary acidic protein; EGFP, enhanced green fluorescent protein; H-100, hypotonic artificial cerebrospinal fluid; 50 mM K+, artificial cerebrospinal fluid with elevated K+ concentration; 3M, 3-month-old animals.

To assess the contribution of the cell somas and processes to the total changes in astrocyte volume, we calculated the ratio of the processes volume change to the volume change of the soma (Figure 7). The exposure to aCSF_H–100_, revealed no significant differences in V_processes_/V_soma_ ratio between the controls and 3xTg-AD mice (Figure 7A). The only significant differences were found in 3M-old animals during washout following the exposure to aCSF_K+_. While swelling of the processes significantly predominated in the controls, in 3xTg-AD mice the ratio approached one, indicating an equal contribution of both cell parts to the total volume changes (Figure 7B). As shown in our previous study (Kolenicova et al., 2020), the volume changes of somas and processes mainly reflect the redistribution of transport mechanisms within the astrocyte membrane. Therefore, we speculate that the increased contribution of processes to the persistent astrocyte swelling in the washout is due to the locally lower expression of channels/transporters responsible for volume regulation. This phenomenon is likely to change during aging, when the expression of the channels/transporters is equally distributed on both somas and processes.

### The Number of Cells That Respond to Pathological Conditions With a Low Rate of Swelling Is Increased in Alzheimer’s Disease

**FIGURE 8:**
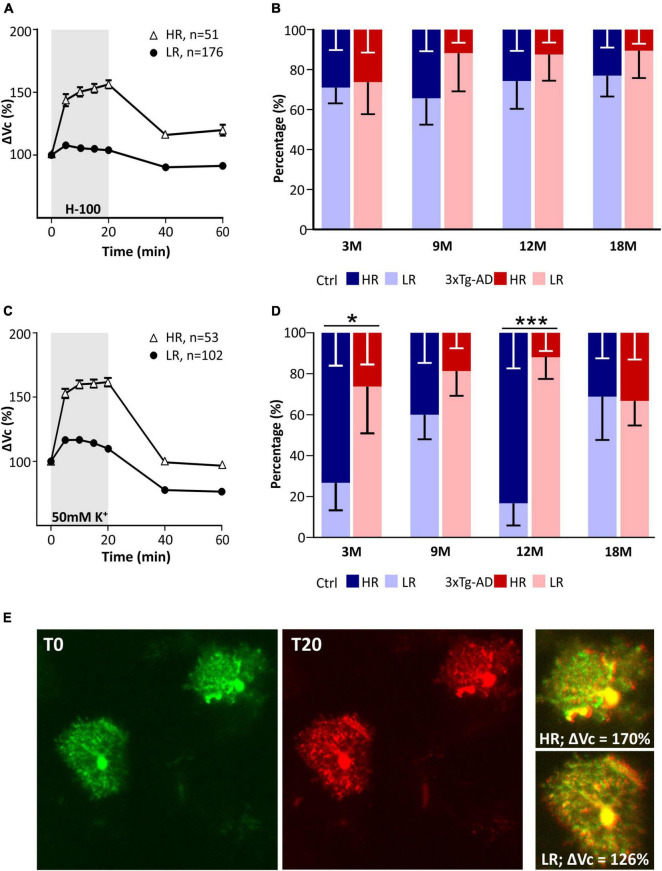
Characterization of two groups of EGFP-positive astrocytes responding to pathological conditions with high or low rate of swelling identified in hippocampus of controls and 3xTg-AD mice. Subpopulations differ in the size of the swelling and the course of volume changes during the exposure to hypo-osmotic stress **(A)** or hyperkalemia **(C).** Comparison of the incidence of HR- and LR-astrocytes between controls and 3xTg-AD in individual age groups in hypo-osmotic stress **(B)** or hyperkalemia **(D)**. **Note** that the number of astrocytes responding with a low rate of swelling is significantly increased in 3- and 12M-old 3xTg-AD mice. **(E)** Representative images of HR- and LR- EGFP-expressing astrocytes before (T0, red) and after the 20-min exposure to 50 mM K^+^ (T20, green). Overlays show merged images of cells with their initial volume (red) and swollen cells (green). Green area represents swelling induced by 50 mM K^+^. Data are presented as mean ± SEM. Statistical significance was determined by two-way ANOVA test with Tukey’s *post hoc* test. Asterisks indicate significant differences (*p < 0.05, ***p < 0.001). Ctrl, control; 3xTg-AD, triple transgenic model of AD crossbred with GFAP/EGFP mice; HR, high-responding astrocytes; LR, low-responding astrocytes; H-100, hypotonic artificial cerebrospinal; 50 mM K+, artificial cerebrospinal fluid with elevated K^+^ concentration; 3M, 9M, 12M, 18M, 3-, 9-, 12-, 18-month-old animals; n, number of cells.

The astrocyte volume changes in all tested groups were analyzed using SOM (self-organizing map) analysis to determine whether distinct astrocyte subpopulations with a different ability to regulate their volume also appear during aging, as described in our previous study (Benesova et al., 2009). Cells from all ages were pooled and divided by SOM analysis into two groups of astrocytes based on the size of their swelling and the course of volume changes over time (Figure 8). These two groups were profiled based on volume changes induced by both hypo-osmotic stress (Figure 8A) and hyperkalemia (Figure 8C). Two groups of astrocytes were labeled following our previous study as HR (high-responding) and LR (low-responding). In hypo-osmotic stress, the volume of HR cells reached ∼156% in the 20th min of aCSF_H–100_ exposure, while the maximal volume of LR cells was only ∼108%. The percentage of HR and LR cells did not differ between the controls and 3xTg-AD mice in either age group (Figure 8B). In hyperkalemia, the volume of HR cells reached the maximal value of ∼162% in the 20th min of aCSF_K+_ exposure, while the maximal volume of LR cells did not exceed ∼117%. The percentage of HR and LR cells differed significantly between the control and 3xTg-AD in 3M and 12M mice (Figure 8D). In 3M controls, the HR group comprised 73% astrocytes, while in the 3M 3xTg-AD mice it was only 26%. In the 12M animals, the HR group was as high as 85% of control astrocytes, while only 18% of 3xTg-AD astrocytes.

Collectively, two subpopulations of astrocytes were present in all age groups in both the controls and 3xTg-AD animals. The only difference in the incidence of HR/LR cells was found in 3M and 12M 3xTg-AD mice during their exposure to aCSF_K+_.

### The Expression of Astrocytic Genes Responsible for the Ion and Water Movement Changes With the Progression of Alzheimer’s Disease Symptoms

As the volume changes of astrocytes exposed to hypo-osmotic stress or hyperkalaemia differed significantly between the controls and 3xTg-AD mice, we hypothesized that the different volume changes reflect the altered expression of channels/transporters involved in the transport of osmotically active substances and water across the astrocytic membranes. Therefore, we performed a scRT-qPCR to determine the expression profiles of astrocytic channels, transporters and receptors that participate in maintaining ion and neurotransmitter homeostasis. The analysis of expression of 96 genes (for their complete list see Supplementary Table 1) was carried out in EGFP-positive cells collected from the CA1 region of the hippocampus from the 3M, 9M, 12M, and 18M controls or 3xTg-AD mice.

**FIGURE 9:**
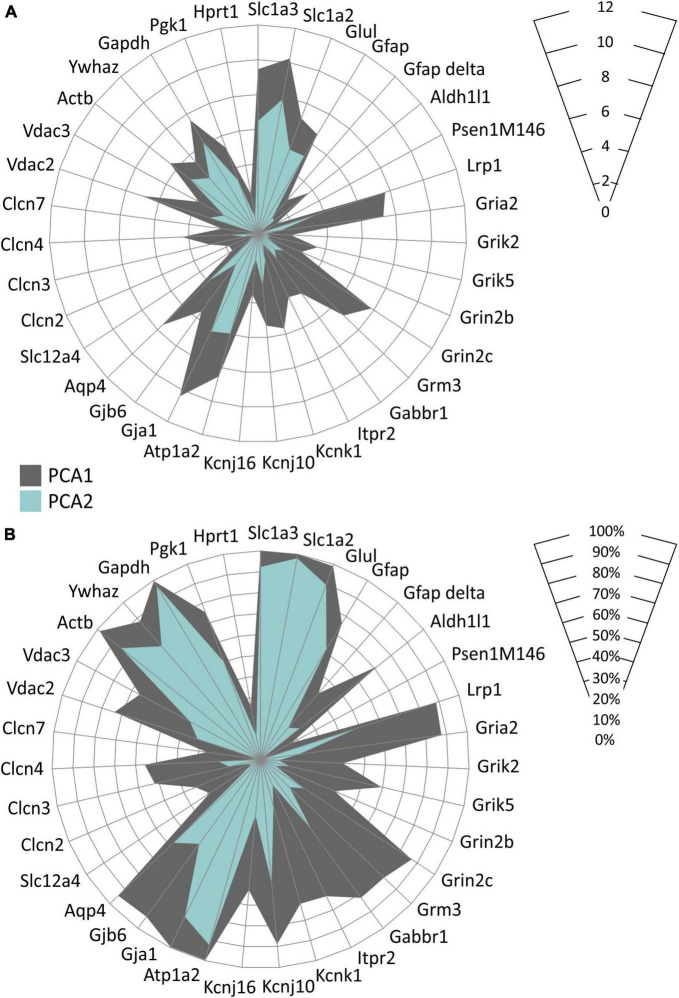
Characterization of two EGFP-positive astrocyte subpopulations differing in the expression of the homeostatic genes identified in hippocampus of controls and 3xTg-AD mice. Subpopulations differ in levels of expression of selected genes **(A)** and in the percentage of cells expressing these genes **(B)**. Only genes, the expression of which was changed significantly (*p* < 0.05) are depicted. Statistical significance was determined by two-way ANOVA test with Tukey’s *post hoc* test.

In accordance with the above-described data, we also found distinct astrocyte subpopulations based on their gene expression profiles. Employing the principal component analysis (PCA) two groups of EGFP-positive astrocytes, PCA1 and PCA2 were identified, differing in the expression levels of 35 genes as well as in the percentage level of cells expressing these genes (Figure 9). Differentially expressed genes comprise, primarily, those encoding aquaporins and connexins (*Aqp4, Gja1, Gjb6*), chloride and anion channels (*Clcn3, Clcn4, Vdac2*), potassium channels (*Kcnk1, Kcnj10*) and metabotropic and ionotropic glutamate receptors (*Grm3, Grin2c, Gria2*).

**FIGURE 10:**
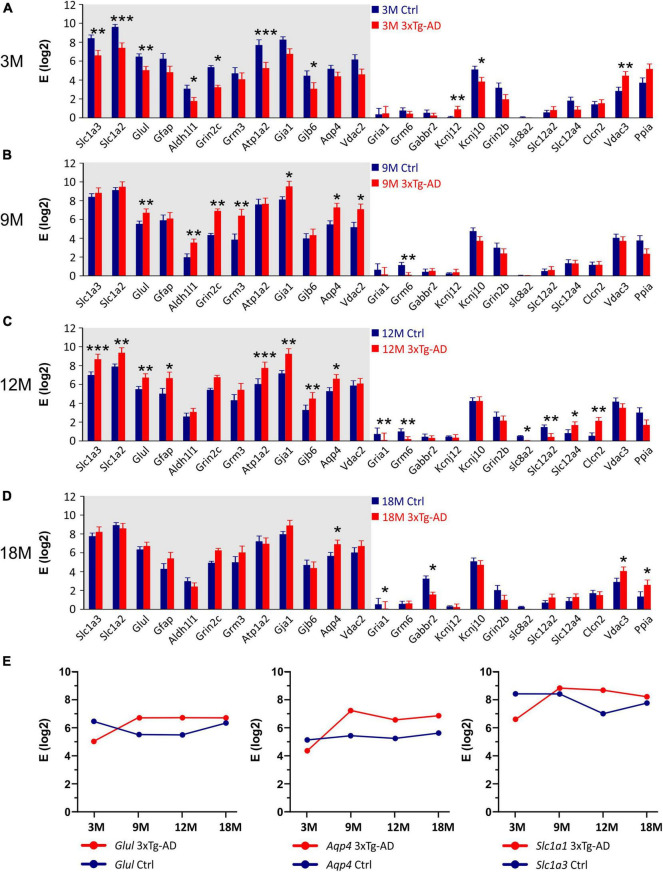
Gene expression analysis in hippocampal EGFP-positive astrocytes of 3xTg-AD mice. **(A–D)** Relative mRNA levels (log scale) of genes significantly changed in 3M- **(A)**, 9M- **(B)**, 12M- **(C)** and 18M- **(D)** -old 3xTg-AD mice compared to age-matched controls. Normalized data are expressed as fold changes compared to the sample with the lowest expression of the gene. Data are presented as mean ± SEM. Statistical significance was determined by two-way ANOVA test with Tukey’s *post hoc* test. Genes undergoing significant changes during aging are indicated by a gray background. Asterisks indicate significant differences (*p < 0.05, **p < 0.01, ***p < 0.001). **(E)** Age dependent changes of relative mRNA levels of genes encoding glutamin synthetase (*Glul*), aquaporine 4 (*Aqp4*) and glutamate transporters GLAST (*Slc1a3*). Ctrl, control; 3xTg-AD, triple transgenic model of AD crossbred with GFAP/EGFP mice; GFAP, glial fibrillary acidic protein; EGFP, enhanced green fluorescent protein; 3M, 9M, 12M, 18M, 3-, 9-, 12-, 18-month-old animals; Aliases of all genes are summarized in Supplementary Table 1.

Comparing the 3xTg-AD mice and their age-matched controls significant differences in the expression levels of 24 genes were found (Figure 10). In the 3M 3xTg-AD mice, most of the differentially expressed genes were downregulated, compared to the controls. The most noteworthy decreases in the gene expression were observed for *Glul* encoding glutamine synthetase (GS), *Grin2c* encoding 2C subunit of NMDA receptors, *Gjb6* encoding connexins 30, *Kcnj10* encoding Kir4.1 potassium channel, *Slc1a2 and Slc1a3* encoding excitatory amino acid transporters (EAATs) and *Atp1a2* encoding α-2 subunit of Na^+^/K^+^ ATPase. In the 3M 3xTg-AD mice the only upregulated astrocytic gene was *Vdac3* encoding voltage -dependent anion channel 3. Notably, the expression of genes, which were downregulated in the 3M 3xTg-AD mice, increased with aging. The maximal difference was observed in the 12M 3xTg-AD mice and their age-matched controls. At 18 months, the expression of most genes was comparable between the controls and 3xTg-AD mice. The age-related expression changes of selected genes are shown in Figure 10E. For other genes involved in astrocyte volume changes, such as *Slc12a2, Slc12a4*, and *Clcn2* (encoding (Na^+^)-K^+^-Cl^–^ cotransporters and ClC2 channels), we observed rather low expression levels in astrocytes from both the controls and 3xTg-AD mice. Nevertheless, in the 12M 3xTg-AD mice, *Slc12a2* was downregulated, while *Slc12a4* and *Clcn2* were upregulated.

Overall, we already observed changes at the gene level in 3M animals, which can affect the uptake of ions and neurotransmitters from the ECS, as well as the ability of astrocytes to regulate their volume. The decreased expression of some genes changes with age, possibly due to compensatory mechanisms. In addition, we found that even within one age group, there are significant differences in the gene expression between astrocytes. Based on this, we identified two groups of astrocytes, differing in the expression levels of 35 genes, as well as in the percentage of cells expressing these genes.

## Discussion

In this study, we described a new mouse strain, created by crossing the 3xTg-AD model of AD (Oddo et al., 2003) with GFAP/EGFP mice (Nolte et al., 2001), in which we detected an intracellular expression of Aβ in pyramidal neurons in 3M animals which is earlier than in the original 3xTg-AD model. In this AD model, we did not observe any changes in the ECS volume fraction during aging, but the increase in the number of obstacles that we observed in physiological aging occurred faster. The changes in α and λ evoked by hypo-osmotic stress were comparable between the AD model and the age-matched controls, the only difference was observed in the rate of α recovery in 9M and 12M animals. Similarly, changes in α and λ evoked by hyperkalemia were comparable between the 3xTg-AD mice and controls within 3 - 12M, however in 18M mice, a marked decrease in α and compromised recovery was detected in controls compared to the 3xTg-AD mice. We can subsequently summarize that single astrocyte swelling evoked by both hypo-osmotic stress and hyperkalemia was lower in the 3xTg-AD mice than in controls and it did not change with increasing age. We further found that these changes only occur in a certain subpopulation of astrocytes, which becomes more numerous during the progression of AD symptoms. Finally, we already observed a decrease in the expression of genes in the 3M animals, which can affect the uptake of ions and neurotransmitters from the ECS, as well as the ability of astrocytes to regulate their volume. These are mainly genes of astrocytic glutamate transporters, α2β2 subunit of Na^+^/K^+^-ATPase, connexin 30 or Kir4.1 channels.

### Animal Model

For studying the morphological changes of astrocytes, we generated a new mouse strain by crossbreeding 3xTg-AD mice with GFAP/EGFP mice (Nolte et al., 2001). The original triple transgenic model was generated by Oddo et al. (2003) to study the interactions between Aβ and tau and their joint effect on the synaptic function. The triple transgenic mice develop both the main features of AD, the amyloid plaques and the neurofibrillary tangles. The authors reported the intraneuronal Aβ immunoreactivity as one of the earliest manifestations of the neuropathology. It was observed in the hippocampus from the age of six months. In contrast, our results already revealed the intracellular Aβ expression in the hippocampal pyramidal neurons in 3M animals. On the other hand, in the original work, the hippocampal expression of extracellular Aβ deposits was observed from 12 months, while we failed to detect them until 18-months of age. A possible explanation for this discrepancy is the different genetic background of the experimental animals. The original 3xTg-AD model has a different genetic background (C57Bl/6) to the GFAP/EGFP model (FVB/NJ), therefore our double transgenic model has a mixed background. A distinct phenotype of AD models created on different genetic backgrounds has been suggested by Frohlich and co-authors (Frohlich et al., 2013). They found a significantly higher number of plaques as well as an increased intracerebral concentration of Aβ42 in APP/PS1 mice on a C57Bl/6J background than in FVB/N background mice (APPtg-FVB). Furthermore, these data correlated with the microglial activity which was enhanced in the APPtg-FVB mice. Qosa and Kaddoumi (2016) described the different clearance of Aβ in four mouse strains widely used for the development of AD mouse models. The authors observed a decreased expression of LRP1 protein, which is responsible for Aβ clearance in SJL/J mice compared to C57Bl/6, FVB/N, and BALB/c. They further reported that the brain degradation of Aβ decreases in BALB/c, FVB/N, and SJL/J mice after administration rifampicin, which is a commonly used antibiotic that has previously been found to increase Aβ clearance across the blood-brain barrier (Qosa et al., 2012). Comparable reduced degradation was not observed in C57Bl/6 mice.

### Changes in the Morphology of Astrocytes and Their Effect on Extracellular Space Diffusion Parameters

A gradual age-related decrease in α and increase in λ in mouse hippocampus with the most profound changes in 18M animals was reported in our recent study (Kolenicova et al., 2020), and confirmed in this study. Apart from the specific brain structures (thalamus, brainstem acoustic nuclei), rich on protective perineuronal and perisynaptic nets of condensed ECM, smaller or larger alterations in brain diffusion detected by the RTI method or diffusion-weighted magnetic resonance seems to be a common feature of aged nervous tissue (Sykova and Nicholson, 2008). Some controversy on the extent of age-evoked α, λ or apparent diffusion coefficient of water (ADC_W_) changes observed among the studies, resulted from complex structural alterations of the aging tissue including changes in glial atrophy and/or astrogliosis (Rodriguez-Arellano et al., 2016), deposits of Aβ (Xekardaki et al., 2015), microglia activation (Harry, 2013), or changes in the ECM composition (Morawski et al., 2012; Morawski et al., 2014; Vegh et al., 2014b), which can affect the ECS diffusion parameters and can vary in distinct brain regions or different species. Interestingly, more substantial α and ADC_W_ decreases in females than in males were revealed during “late” aging (17-28- months of age) (Sykova et al., 2005) while no gender-related differences were detected in “early” aging (16-18 months of age) (Cicanic et al., 2018).

In contrast to physiological aging, we detected no age-related decline in α in 3xTg-AD mice. This finding corresponds with the observation in another AD model, APP23 mice (Sykova et al., 2005) and may be associated with amyloid deposits in the ECS, preventing the shrinkage of the ECS volume in aged tissue. However, the immunohistochemical analysis showed an extracellular amyloid load no earlier than in the 18M 3xTg-AD animals (Figure 1). Therefore, the contribution of other factors such as atrophy of astrocytes (Rodriguez-Arellano et al., 2016) or shifts in the composition of ECM (Morawski et al., 2012; Sethi and Zaia, 2017) should be taken into account, especially in the earlier phase of aging.

Sykova et al. (2005) also reported a decrease in ADC_W_ and ADC_TMA_ indicating more hindered brain diffusion than in the controls, but only in APP23 females and not in APP23 males. Moreover, some studies showed a gender-dependent impairment of water-maze performance in aged APP23 mice, as well as a higher plaque load in females than in males (Kelly et al., 2003; Sykova et al., 2005). On the contrary, in 3xTg-AD mouse model containing mutations not only in APP but also in tau protein and presenilin, we did not detect any gender-related difference in amyloid load, the ECS diffusion parameters, or in astrocyte swelling. In this study, we observed more hindered extracellular diffusion (larger λ values) in the 12M 3xTg-AD mice than in the age-matched controls. In fact, an increase in λ during physiological aging was accelerated in the 3xTg-AD mice with a substantial “jump” already between the 9 and 12 months of age, while a similar substantial λ increase occurred between 12 and 18 months of age in the controls. Again, as amyloid is not expressed extracellularly in 12M animals, other key elements creating diffusion barriers, such as morphological changes of glia cells and/or increase of ECM expression, have to play a role in the observed λ increase.

Our previous studies of various pathological conditions revealed that increased ECS volume and/or tortuosity are in accordance with the ECM overexpression (Roitbak and Sykova, 1999; Zamecnik et al., 2004), while smaller values of α and/or λ associate with overall decrease of ECM content or lack of a core ECM molecules (Sykova et al., 2005; Bekku et al., 2010). Thus, to explain increased values of both α and λ in the aged 3xTg-AD mice, we would expect ECM overexpression in AD-affected tissue. In contrast, a loss of ECM forming perineuronal nets in AD tissue was reported in older studies based on detection of N-acetylgalactosamine by lectins Wisteria foribunda and vicia villosa (Baig et al., 2005). However, more recent study of Morawski et al. (2012) showed that this loss of ECM was an artifact caused by decomposition of post mortem tissue. In fact, perineuronal nets remained intact in well-preserved AD tissue and hyaluronan is even enriched in amyloid plaque territories (Morawski et al., 2012). Similarly, significant increases in the levels of several protein components of the ECM, with increases in Aβ levels was found in the hippocampus; these increases occurred before the onset of plaque formation in association with impairment of long-term potentiation and contextual memory (Vegh et al., 2014a).

Progressive increase in tortuosity with aging in 3xTg-AD mice without changes in the ECS volume might seem counterintuitive but only in comparison with acute states, such as acute ischemia (Vorisek and Sykova, 1997). In this case, cell swelling is accompanied by a compensatory shrinkage of the ECS leading to a decrease in α and increase in λ due to a higher concentration of already existing diffusion barriers in a smaller space. However, we have shown in several of our previous studies that α and λ behave independently in chronic or long-lasting states where the amount of diffusion barriers is increased (Roitbak and Sykova, 1999; Zamecnik et al., 2004; Anderova et al., 2011) or decreased (Bekku et al., 2010; De Santis et al., 2020). Generally, there are three main sources of diffusion barriers: 1. fine cell processes, especially astrocytic, 2. macromolecules of ECM and 3. dead space microdomains, i.e., concave geometrical formation of the ECS, where diffusing molecules can be “trapped” (for review see Hrabetova and Nicholson, 2004; Sykova and Nicholson, 2008; Wolak and Thorne, 2013). Increased tortuosity in aged 3xTg-AD presumably results from a combination of all these factors. Both AD-related reactive astrogliosis with morphological rebuilding of astrocytic processes and increased expression levels of several ECM molecules were described in the very late stages of AD development (Morawski et al., 2012; Vegh et al., 2014a; Rodriguez-Arellano et al., 2016). Contribution of dead spaces is also possible especially in late stages of AD, when geometry of the ECS can be altered by the presence of amyloid plaques.

In contrast to the reduced hyperkalemia-induced swelling of astrocytes in the 9M and 18M 3xTg-AD mice in comparison with the controls, we did not detect similar differences in the ECS diffusion parameters. When comparing the extent of volume changes, we had to evaluate the relative changes due to different basal values α and λ between the control and 3xTg-AD mice, especially in the ages of 12 and 18 months. Relative values of volume fraction and tortuosity, calculated when the control values were set to 100%, did not show any significant differences between the control and 3xTg-AD mice, neither after hypotonic stress nor severe hyperkalemia. In animals with physiological aging, the exposure to 50 mM K^+^ evoked more profound changes of absolute α in the 3M and 12M mice than in the 9M and 18M groups, similarly to changes in astrocyte swelling. In contrast, the pattern of α changes in 3xTg-AD animals was similar in all the tested age groups. In fact, there was a trend to a larger and especially faster α decrease during 50 mM K^+^ exposure in the 3xTg-AD mice in comparison with the controls (Supplementary Figure 3). However, the differences became statistically insignificant after post-test. The discrepancy between the ECS volume changes and astrocyte swelling was already observed in our previous studies (Dmytrenko et al., 2013; Anderova et al., 2014; Kolenicova et al., 2020). It is quite predictable, as the changes of α measured by the RTI method reflect volume changes in all cell types, not just in astrocytes, and average thus the values from about 0.01-0.001 mm^3^ of tissue. Apart from astrocytes, other cells with a large capability of swelling are neurons. A recent study by Genocchi et al. (2020) used a computational model of cortical spreading depression to explore how different parameters, including changes in AQP4 channel expression in astrocytes, neuron/astrocyte ratio and activity of Na^+^/K^+^-ATPase or chloride channels affect the swelling of neurons and astrocytes. Their results show that changes in each of these parameters may shift the proportion between neuronal and astrocytic swelling (Genocchi et al., 2020). Our results of astrocytic gene profiling undoubtably showed significant differences in the expression of some of the above-mentioned genes. We can therefore assume that a proportion of swelling of neurons and astrocytes (and possible other cell types) in the 3xTg-AD mice, differs from that in the control animals and may contribute to the larger discrepancy in the findings obtained by RTI and 3D confocal morphometry.

Following the evoked cell swelling, the values of α only recovered fully to the basal conditions in 3M, 9M, and 12M control animals and 3M and 9M 3xTg-AD mice, after aCSF_H–100_. The recovery in the older groups was only partial. The partial recovery was also detected in all the tested groups after the application of aCSF_K+_, with the exception of the 18M control animals, where no recovery was detected. This is in contrast to the astrocytic volume measurements, where a full recovery or even undershoot was observed in all the tested groups after hypo-osmotic stress or hyperkalemia. This discrepancy is due to the contribution of other cell types to the changes of α measured by the RTI method. Most other studies only attribute the ability to recover cell volume to astrocytes. Studies in neuronal cultures showed some extent of volume regulation in neurons by the releasing of taurine (Olson and Li, 2000; Li and Olson, 2008). However, a more recent study showed that neurons in tissue slices during oxygen/glucose deprivation swell rapidly, but are not capable of returning their volume to the original value (Risher et al., 2009). Astonishingly, in the 18M 3xTg-AD mice, the recovery was partial, while no recovery was observed in the age-matched control mice. No recovery in the control aged animals suggest possible irreversible tissue damage by severe depolarization evoked by 50 mM K^+^, which could be even more pronounced in the older animals with an already small basal ECS volume. In fact, in 18M control mice, 50 mM K^+^ evoked an α decrease to 0.07-0.08 which is close to values reached in terminal ischemia-anoxia (Vorisek and Sykova, 1997). In such a small space, concentration of glutamate or other toxic substances diluted in the interstitial fluid can be extremely high and may evoke irreversible cytotoxic damage of the tissue. Studies concerning spreading depression (SD), which main feature is a high potassium-evoked depolarization, reported prolonged SD duration and hampered recovery from SD in aged brain in comparison with young one and related these finding to functional alterations in membrane transporters and channels (for review see Hertelendy et al., 2019). Age-related impairment of ionic and neurotransmitter uptake was demonstrated also here and in our previous study (Kolenicova et al., 2020). Assuming that ionic and neurotransmitter homeostasis is altered in both control and 3xTg-AD aged mice, the larger initial ECS volume in the aged 3xTg-AD can be a protective factor buffering the concentration changes of toxic substances and enabling at least a partial recovery to control values.

### Single Astrocyte Volume Changes and Their Relationship to Expression Levels of Channels and Transporters

In our recent study (Kolenicova et al., 2020), we showed that single astrocyte volume changes under exposure to high K^+^ concentration alter during aging, which is a natural neurodegeneration process. Here we compare the age-related changes with changes caused by the pathological processes typical of AD. We show that the changes observed during aging occur earlier in the 3xTg-AD model of AD. We found lower volume changes in these mice when compared to the controls and these changes did not alter among the age groups. On the other hand, volume changes induced by hypo-osmotic stress showed no differences related to aging (Kolenicova et al., 2020), but in the 3xTg-AD astrocytes we observed significantly lower swelling than in the controls, similarly, to hyperkalemia. These results suggest that the mechanisms responsible for the uptake or extrusion of osmotically active substances, i.e., ions and neurotransmitters, as well as water transport mechanisms are impaired in the 3xTg-AD mice. We hypothesized that these defects are due to the altered expression of channels and transporters previously associated with astrocyte swelling under hypo-osmotic stress or ischemic conditions. Therefore, we performed sc RT-qPCR analysis and compared the gene expression of 96 selected homeostatic genes (Supplementary Table 1). In general, even in the young, 3M 3xTg-AD mice, a significantly lower expression of some homeostatic genes was observed compared to the control. In the 9M 3xTg-AD mice, the expression of these genes increased and remained at the same level until the age of 18 months. We believe that an overexpression may be a compensatory mechanism in response to the deterioration of astrocyte homeostatic functions.

One of the transporters for which we predicted changes in the expression profile was the Na^+^-K^+^-Cl^–^ cotransporter (NKCC1). In the previous study (Kolenicova et al., 2020) we showed that astrocyte volume changes during exposure to hyperkalemia were inhibited by bumetanide, an NKCC1 blocker. This cotransporter was also described to be involved in K^+^-induced astrocyte swelling *in vitro* (Su et al., 2002) or in the optic nerve (MacVicar et al., 2002). NKCC1 contributes to the cell swelling by transferring ions into the cells and also by co-transferring water along with its substrates (Macaulay and Zeuthen, 2012). Since NKCC1 is abundantly expressed on other cell types in addition to astrocytes, such as endothelial cells which may also contribute to the cerebral oedema formation, the inhibition of this transporter is one of the options considered for the treatment of cerebral oedema (Deng et al., 2016). On the other hand, some authors report that astrocytic swelling induced by neuronal activity or 10.5 mM concentration of extracellular K^+^ is not due to NKCC1 activity (Larsen et al., 2014; Walch et al., 2020). However, the sc RT-PCR analysis of astrocytes isolated from the 3xTg-AD mice revealed no significant change in the *Slc12a2* (NKCC1 encoding gene) expression, which is consistent with the literature, as no reference was found to the fact that Na^+^-K^+^-Cl^–^ cotransporter expression was altered in AD models. Therefore, we believe that it is unlikely that NKCC1 expression impairment is the cause of reduced swelling under the exposure to hypo-osmotic stress or hyperkalemia.

We further focused on the changes in the expression of AQP4 as this water channel plays multiple roles in the pathophysiology of AD. AQP4 affects ion and glutamate homeostasis, synaptic plasticity, astrocytic Ca^2+^ signaling, neuroinflammation, as well as Aβ and tau protein clearance (Lan et al., 2016; Assefa et al., 2018). Changes in AQP4 expression have even been considered a cause of the accumulation of Aβ plaques (Peng et al., 2016) and tau protein (Harrison et al., 2020) in the brain parenchyma (Tarasoff-Conway et al., 2015; Valenza et al., 2019). The accumulation is caused by the disruption of the so-called glymphatic system, which is responsible for the clearance of excess or defective proteins from the ECS (Plog and Nedergaard, 2018; Reeves et al., 2020). Since most studies describe an increase in AQP4 expression, the decreased clearance is possibly due to the loss of polarization, redistribution of AQP4, and mislocalization on non-endfoot membrane, rather than an overall change in expression. Changes in the localization of AQP4 on the astrocyte membrane have been described in the tg-ArcSwe mouse model of AD (Yang et al., 2011). The authors reported a loss of AQP4 from endfoot membranes at sites of perivascular amyloid deposits, emerging just after the appearance of the first plaques. Moreover, they found an upregulation of AQP4 in the neuropil surrounding the plaques. The increased expression was also observed in patients with AD or cerebral amyloid angiopathy by Moftakhar and co-authors (Moftakhar et al., 2010). Similarly, Hoshi and co-authors (Hoshi et al., 2012) showed an increased AQP4 expression in the temporal lobe of patients with sporadic or familiar form of AD; however, the expression differed between the inferior, middle and superior temporal cortices. Moreover, the authors report that the increased expression of AQP4 in senile plaques occurred during early Aβ deposition, whereas AQP4 downregulation occurred in the later stage of Aβ plaque formation. The role of AQP4 in astrocyte swelling and brain oedema formation has been suggested in several studies in which a less swelling in mice with deleted AQP4 was observed (Manley et al., 2000; Papadopoulos and Verkman, 2005). Another experimental approach used modifications in the subcellular distribution of AQP4 molecules, induced by the deletion of dystrophin or α-syntrophin in Syn-/- mice, leading to the reduced expression of AQP4 localized in the perivascular membranes of astrocytic endfeet. A delayed brain oedema formation after water intoxication was observed in these transgenic mice (Vajda et al., 2002; Amiry-Moghaddam et al., 2004). On the other hand, the participation of AQP4 in the swelling of individual astrocytes has been confirmed to be neither in elevated K^+^ nor in hypo-osmotic stress (Murphy et al., 2017; Walch et al., 2020). In the first study the authors observed an increased swelling of astrocytes in hippocampal slices of AQP4–/– mice compared to the controls in a hypoosmolar cell swelling model (Murphy et al., 2017). In the second study, the authors observed no differences between the swelling of AQP4–/– mice and controls and slower recovery to baseline volume during the washout period after the 10.5 mM K^+^ application (Walch et al., 2020). Therefore, they hypothesized that AQP4 is dispensable for astrocyte swelling, and in the absence of AQP4, astrocytes have impaired volume recovery. Their results are in line with findings that the deletion of AQP4 increased ECS shrinkage, indicating a possible role for AQP4–/– in astrocyte volume regulation (Haj-Yasein et al., 2012). Finally, a study with the new isoform-selective AQP4 inhibitor TGN-020 showed that K^+^ uptake and associated astrocytic swelling are not dependent on AQP4 function (Toft-Bertelsen et al., 2021). All of the above-mentioned facts are consistent with the results presented in this work. In the 3xTg-AD model of AD, we observed a significant increase in AQP4 mRNA expression, along with an increasing incidence of Aβ plaques. These results are in slight contrast to Bronzuoli and co-workers (Bronzuoli et al., 2019), who reported an increase in AQP4 protein expression in the hippocampus of the 3xTg-AD mice during aging, but regardless of genotype. This discrepancy may stem from the different approaches used in the two studies, since we determined the mRNA expression at the single-cell level and in a subpopulation of EGFP-labeled astrocytes, while Bronzuoli et al. determined the total protein expression by Western blotting or immunohistochemistry. As AQP4 does not appear to play a role in astrocyte swelling, but rather contributes to cell volume regulation, increases in its expression correspond to lower swelling in the 3xTg-AD mice compared to the controls.

Other mechanisms that attracted our attention were those related to glutamate homeostasis. One of the basic properties of astrocytes, which are impaired in AD, is the uptake of glutamate from ECS. Several studies have revealed significant changes in the expression of glutamate transporters in both AD patients and mouse models (Jacob et al., 2007). On the contrary, other studies have not confirmed the reduced expression of EAATs (Beckstrom et al., 1999; Kulijewicz-Nawrot et al., 2013). The altered expression of glutamate-aspartate transporter (GLAST) has been observed by Ikegaya et al. (2002), Abe and Misawa (2003), Jacob et al. (2007), Matos et al. (2008). While Abe and Ikegaya observed GLAST reduction the others published opposite results. The conflicting data can be explained by different methodological approaches including amyloid preparation or culture conditions, but also by internalization or mislocalization as shown by Scimemi and co-authors (Scimemi et al., 2013) for glutamate transporter 1 (GLT-1). The authors reported an impaired glutamate clearance due to mislocalization of GLT-1 expression in slices treated with monomeric or oligomeric Aβ 1-42. In addition, regional differences such as those mentioned by Jacob and co-authors, must also be considered (Jacob et al., 2007). The reduced expression of glutamate transporters results in an impaired glutamate uptake and increased extracellular glutamate levels. This in turn leads to the overactivation of neuronal glutamate receptors resulting in synaptic dysfunction, which significantly contributes to the AD pathophysiology. Here we observed the reduced expression of *Slc1a2* (encoding GLT-1), but only in the 3M animals. In the older transgenic animals, GLT-1 expression values exceeded those observed in the controls, but the differences were rather due to the expression changes occurring in the controls during aging. The same pattern was observed for *Slc1a3* (encoding GLAST) expression.

In addition to the above-mentioned functions of AQP4, this water channel also regulates the function and expression of other channels/transporters, namely glutamate transporters, potassium channels and connexins. An association between AQP4 and GLT-1 has been proposed based on the findings that AQP4 deficiency downregulated GLT-1 in primary cultured astrocytes (Zeng et al., 2007) or mouse amygdala (Li et al., 2012). The changes in amygdala adequately influenced the synaptic plasticity as well as the fear memory (Li et al., 2012). Yang and co-authors (Yang et al., 2013) showed that GLT-1 stimulator (ceftriaxone, a β-lactam antibiotic known to upregulate GLT-1 in astrocytes) improved memory function in AQP4 deficient mice. Moreover, Hinson and co-authors suggested that AQP4 and GLT-1 co-exist in astrocytic membranes as a macromolecular complex, allowing their functional coupling (Hinson et al., 2008). Co-expression of the GLAST with AQP4 was discovered in the study of Nielsen (Nielsen et al., 1997). In line with these findings, we observed a very similar expression pattern for *Slc1a2*, *Slc1a3* and *Aqp4*.

Further, we assumed that the changes in glutamate transporter expression may also affect astrocyte swelling during exposure to pathological conditions. Pathological glutamate levels may contribute to the swelling by influx of Na^+^, H^+^ and glutamate in exchange for K^+^. Nevertheless, the increased levels of intracellular Na^+^ most likely inhibit this type of transport and GLAST and GLT-1 may rather contribute to the volume decrease by the supposed “uptake reversal” (Jabaudon et al., 2000). Moreover, the astrocytic water transport at the pial and vascular interfaces is predominantly mediated by AQP4, whereas the water passage at the neuropil facing membranes is determined by GLTs (MacAulay et al., 2002). Based on the expression changes observed in our study, glutamate transport in younger animals contributes to the astrocytic swelling, as it was upregulated in the control mice that swell more. On the other hand, in the 12M mice, where the GLAST and GLT-1 are upregulated in 3xTg-AD, the reverse transport appears to occur, as the astrocytes swell less. However, these proposals are only speculative, and a deeper examination of these mechanisms is necessary.

In addition to glutamate transporters, the glutamate homeostatic system is also affected by GS, and changes in its function may contribute to cognitive deficits in the diseased brain. Changes in GS expression have been described by Olabarria in 3xTg-AD mice (Olabarria et al., 2011) and by Hoshi in AD patients (Hoshi et al., 2018). In contrast, another human study showed increased levels of GS in the prefrontal cortex in AD (Burbaeva et al., 2005). Our observation is also not in line with Olabarria and co-authors. While these authors observed a GS expression decrease in the dentate gyrus in the 12M and 18M animals and in CA1 in 18M animals, we only detected a decrease in the 3M animals, but the GS expression was slightly increased compared to controls in older animals.

Similar to glutamate transporters, connexins (Cxs) also work in association with AQP4. Connexins form supposed “gap junctions,” through which astrocytes are connected into a network called an astrocytic syncytium. This interconnection of astrocytes enables the effective buffering of K^+^ and glutamate. An example of Cx-AQP4 collaboration is astrocytic K^+^ siphoning, in which increased K^+^ at the site of neuronal activity is taken up by K^+^ channels along with water and shifted towards perivascular cells through astrocytic syncytium. At the end of this process, ions and water are extruded into the bloodstream via ion channels and AQP4 (Nicchia et al., 2005). Connexins play roles in the formation of glioma-induced brain oedema (Li et al., 2015) as well as cerebral oedema following ischemic brain injury (Chu et al., 2017). Bronzuoli and co-authors reported that Cx43 decreased with age in 3xTg-AD mice as well as in controls. Both the Western blotting and immunohistochemical analysis led to the conclusion that the Cx43 expression in the 3xTg-AD mice does not change due to genetic mutation, hence the presence of Aβ and tau in the ECS, but due to aging (Bronzuoli et al., 2019). On the contrary, we show here that the expression of both major astrocyte Cxs, Cx30 and Cx43 (encoded by *Gjb6* and *Gja1*) increased along with the development of the AD symptoms in the 3xTg-AD mice. However, Cxs have been proven to be involved in the cell volume regulation (Li et al., 2004; Ye et al., 2009). Thus, we can speculate that, similarly to AQP4, Cxs contribute to the volume regulation of astrocytes in 3xTg-AD mice.

Remarkable differences in gene expression were also observed for *Atp1a2* (encoding α2β2 isoform of Na^+^/K^+^-ATPase). While in the controls its expression decreased slightly with age, in the 3xTg-AD mice it increased and in the 18M mice the expression in both strains reached a similar level. As the α2β2 isoform is most sensitive to extracellular K^+^ concentration, it provides a route for K^+^ influx into astrocytes that could couple to water movement (Larsen et al., 2014). Therefore, the downregulation of *Atp1a2*, as observed by the gene expression analysis of this study, may contribute to the lesser swelling in the 3xTg-AD mice. Moreover, it possibly leads to breakdown of the electrochemical gradient, which is a prerequisite for neuronal excitability and the activity of glutamate transporters, such as GLAST and GLT-1. Thus, the decreased expression of *Atp1a2* leads secondarily to increased extracellular concentration of glutamate and likewise contributes to glutamate excitotoxicity (Klemens et al., 2019; Belov Kirdajova et al., 2020).

Since we performed the sc RT-qPCR analysis in astrocytes which has been sorted based on the EGFP expression, which was under the control of the GFAP promoter, we suggest that these cells may represent a minor subpopulation of astrocytes that exhibit different expression changes than those found in the other above-mentioned studies. This hypothesis may be supported by the fact that Aβ-induced expression changes are often observed in astrocytes showing reactive features, such as morphological changes as well as an increased GFAP expression. None of these properties were observed in EGFP-positive astrocytes in the hippocampus of the 3xTg-AD mice. Using single-nucleus RNA sequencing Habib and co-authors identified six astrocytic populations in the hippocampus of 5xFAD mice (Habib et al., 2020). The populations were described as low-GFAP, high-GFAP, disease-associated astrocytes (DAA) with transitional-like intermediate states between them. While low-GFAP and high-GFAP astrocytes were identified in both the controls and 5xFAD mice, DAAs were only found in the AD model. DAAs were characterized by the expression of reactive markers such as Serpina3n and Vimentin, and they also showed an increased expression of pan-reactive and inflammation astrocyte signatures. They were mainly found in the vicinity of Aβ plaques and their percentage increased sharply during the aging of the 5xFAD mice. The percentage of low-GFAP astrocytes increased with age in the controls and decreased in the 5xFAD mice, while the percentage of high-GFAP astrocytes decreased in the controls and remained constant in the 5xFAD. Regarding the homeostatic genes analyzed in our study, the largest differences were observed between the low-GFAP and high-GFAP populations of astrocytes. In the low-GFAPs, the genes *Gja1, Aqp4, Slc1a2, Kcnj10, and Kcnk1* were significantly downregulated, while *Grin2c, Gria2, and Slc1a3* upregulated compared to the high-GFAPs. Although our sc RT-qPCR analysis showed two populations of astrocytes with lower/higher GFAP expression, these groups do not correspond to the groups found by Habib et al. First, in our population with low GFAP expression, all other differentially expressed genes are also downregulated; second, the incidence of cells belonging to PCA1 and PCA2 did not change over time as in the study by Habib et al. In order to better classify our group of astrocytes in accordance with this classification, a more detailed analysis of marker gene expression is necessary.

In our previous publications we described the existence of two populations (HR- and LR- astrocytes) of EGFP-positive astrocytes based on the different levels of swelling under exposure to hypo-osmotic stress (Chvatal et al., 2007b) or oxygen-glucose deprivation (Benesova et al., 2012). We proposed that the different swelling mirrored differences in the expression of Cl^–^ and K^+^ channels and Na^+^-K^+^-Cl^–^ cotransporter. Here we show that there are two groups of EGFP-positive astrocytes differing in the expression levels of 35 genes divided by the principle component analysis. These genes include Clcn2 (a gene for the ClC2 channel), which has been reported to be activated by cell swelling (Nielsen et al., 1997) and involved in the RVD by removing Cl^–^ from the cells. In the study of Benesova at al., *Clcn2* was highly expressed in astrocytic subpopulation 1 (comprising LR-astrocytes), thus possibly contributing to their increased ability of volume regulation. Regarding K^+^ channels, we previously suggested that the expression of Kir4.1, TWIK1 and TREK1 may be another reason for the existence of HR- and LR astrocytes. We show here that the genes underlying the PC1 and PC2 subpopulations include *Kcnj10 and Kcnk1* (encoding Kir4.1 and TREK-1 channels). As Kir4.1 has been demonstrated to participate in RVD rather than the swelling (Dibaj et al., 2007; Hirrlinger et al., 2008; Obara-Michlewska et al., 2011), its higher level in the PCA1 population indicates a correlation of PCA1 with cells that better regulate cell volume. Other genes that are also increased in the PCA1 group, namely Cxs and AQP4, correspond to the hypothesis that it is a population of cells with better volume regulation, i.e., LR-astrocytes, as previously proposed (Benesova et al., 2012). The division of the cells into two populations in the present study was performed based on the differential expression of 35 genes. Surprisingly, the population divide was based on the higher/lower expression of all the genes. This may indicate a reduced transcriptional activity in one group of cells. Further investigation of the heterogeneity of EGFP-positive astrocytes in the 3xTg-AD model of AD is therefore necessary. However, it should be also emphasized that transcript levels are not sufficient to predict levels of functional proteins. The level of protein expression can be modulated at the level of translation, protein’s half-life, protein synthesis delay as well as protein transport (reviewed for example by Liu et al., 2016, or Sonneveld et al., 2020). Therefore, we are aware that a more detailed analysis of the expression of the proteins of interest and their localization may better elucidate the changes we observe at the level of changes in astrocyte volume and diffusion parameters of the ECS.

Taken together, in younger age groups of AD mice (3-9M), intracellular Aβ accumulation in hippocampal neurons alone has no effect on neural tissue diffusivity, as α and λ are comparable to age-matched controls. Simultaneously, changes in ECS volume fraction and its recovery are almost comparable at these ages regardless of employed pathological stimulus. Nevertheless, the astrocyte swelling induced by hyperkalemia is markedly reduced in 3xTg-AD mice already at 3M, which might be partly caused by increased incidence of LR-astrocytes or already decreased expression of glutamate transporters, connexin 30 and Kir4.1 at this age. In 9M-old astrocytes from 3xTg-AD mice the expression levels of above mention genes are comparable to controls, which may also fit well with their similar swelling. Hypotonicity-evoked swelling in astrocytes from 3xTg-AD mice was significantly smaller compared to age-matched control possibly due to slightly increased number of LR-astrocytes and increased expression of connexin 43 and AQP4. The discrepancy in recovery of astrocyte volume and the ECS volume fraction after hyposmolality or hyperkalemia evoked cell swelling is obvious in older age groups (12M and 18M). This discrepancy is highly probable due to the shift in the proportion of swelling of cells that can regulate their volume (astrocytes) and cells which are not capable of regulation (neurons and possibly other types) (Risher et al., 2009). Genocchi et al. (2020) calculated that this shift can be induced by alterations in expression of specific channels involved in volume and ionic homeostasis. In 12M 3xTg-AD mice, the recovery of α following hypo-osmotic stress was significantly smaller compared to age-matched controls. We hypothesize that such less efficient recovery of ECS volume fraction after hypo-osmotic stress may originate from a more severe impairment of homeostatic functions in 3xTg-AD than in control mice indicating a faster decline of astrocyte homeostatic functions in AD-affected tissue and larger proportion of neuronal swelling. In 18M mice, homeostatic functions are comparably impaired in both control and 3xTg-AD mice and α recovery is only partial in both groups. In contrast, more effective recovery of α occurred in 18M 3xTg-AD hippocampus following hyperkalemia compared to age-matched control. This opposite trend can be explained by distinct mechanisms underlying cell swelling during both insults together with the larger initial ECS volume in 3xTg-AD mice, in which toxic substances/ions are more diluted and do not cause an irreversible cell damage. Irreversible changes may occur especially in non-astrocytic cell types as we observed no difference in astrocyte swelling between control and 3xTg-AD in 18M animals. For comparison see Supplementary Figure 4.

## Conclusion

Overall, in the present study we have shown that APP Swedish, MAPT P301L, and PSEN1 M146V mutations in the murine model of AD lead to the structural changes in the ECS differing from those observed in physiological aging. We suggest that these changes are caused by cell atrophy on the one hand, and shifts and alterations in the ECS content, including an increase in the diffusion obstacles (barriers), on the other. However, these changes do not influence ECS shrinkage under exposure to hypo-osmotic stress or hyperkalemia, but they do affect the ECS recovery during the washout.

Larger ECS and increased tortuosity further affect the function of astrocytes, especially the uptake of ions and neurotransmitters. We have also shown that astrocytes in 3xTg-AD mice swell less during the exposure to hypo-osmotic stress or hyperkalemia, when compared to controls. However, this difference is also partially caused by the higher incidence of cells that swell very little or not at all. We therefore propose that this group of astrocytes expresses a higher number of channels involved in RVD or fewer ion channels/transporters participating in ion/neurotransmitter uptake. Considering age-related changes in tortuosity and astrocyte volume swelling/regulation, we conclude that AD can be understood as an accelerated form of physiological aging even at the level of basic homeostatic functions of astrocytes, such as ion/neurotransmitter uptake and water management.

## Data Availability Statement

The original contributions presented in the study are included in the article/Supplementary Material, further inquiries can be directed to the corresponding author/s.

## Ethics Statement

The animal study was reviewed and approved by Institute of Experimental Medicine ASCR Animal Care Committee, Prague, Czechia.

## Author Contributions

JT and MK analyzed and interpreted the data, wrote the manuscript, and prepared all figures. DK, TF, ZH, BP, LM, and JK performed 3D morphometry, IHC, prepared cell suspensions and performed FACS. MK and MC performed real-time iontophoretic method. LuV, PA, and DZ performed RT-qPCR measurement, processed and analyzed data. MA and LyV conceived and supervised the study. All authors contributed to the article and approved the submitted version.

## Conflict of Interest

The authors declare that the research was conducted in the absence of any commercial or financial relationships that could be construed as a potential conflict of interest.

## Publisher’s Note

All claims expressed in this article are solely those of the authors and do not necessarily represent those of their affiliated organizations, or those of the publisher, the editors and the reviewers. Any product that may be evaluated in this article, or claim that may be made by its manufacturer, is not guaranteed or endorsed by the publisher.
