# Supplementary material for: Compromised Astrocyte Swelling/Volume Regulation in the Hippocampus of the Triple Transgenic Mouse Model of Alzheimer’s Disease
Source: Front Aging Neurosci. 2022 Jan 27;13:783120. doi: 10.3389/fnagi.2021.783120 (PMC8829436; doi:10.3389/fnagi.2021.783120)
Supplement: Supplementary file 1 [file Image_1.pdf]

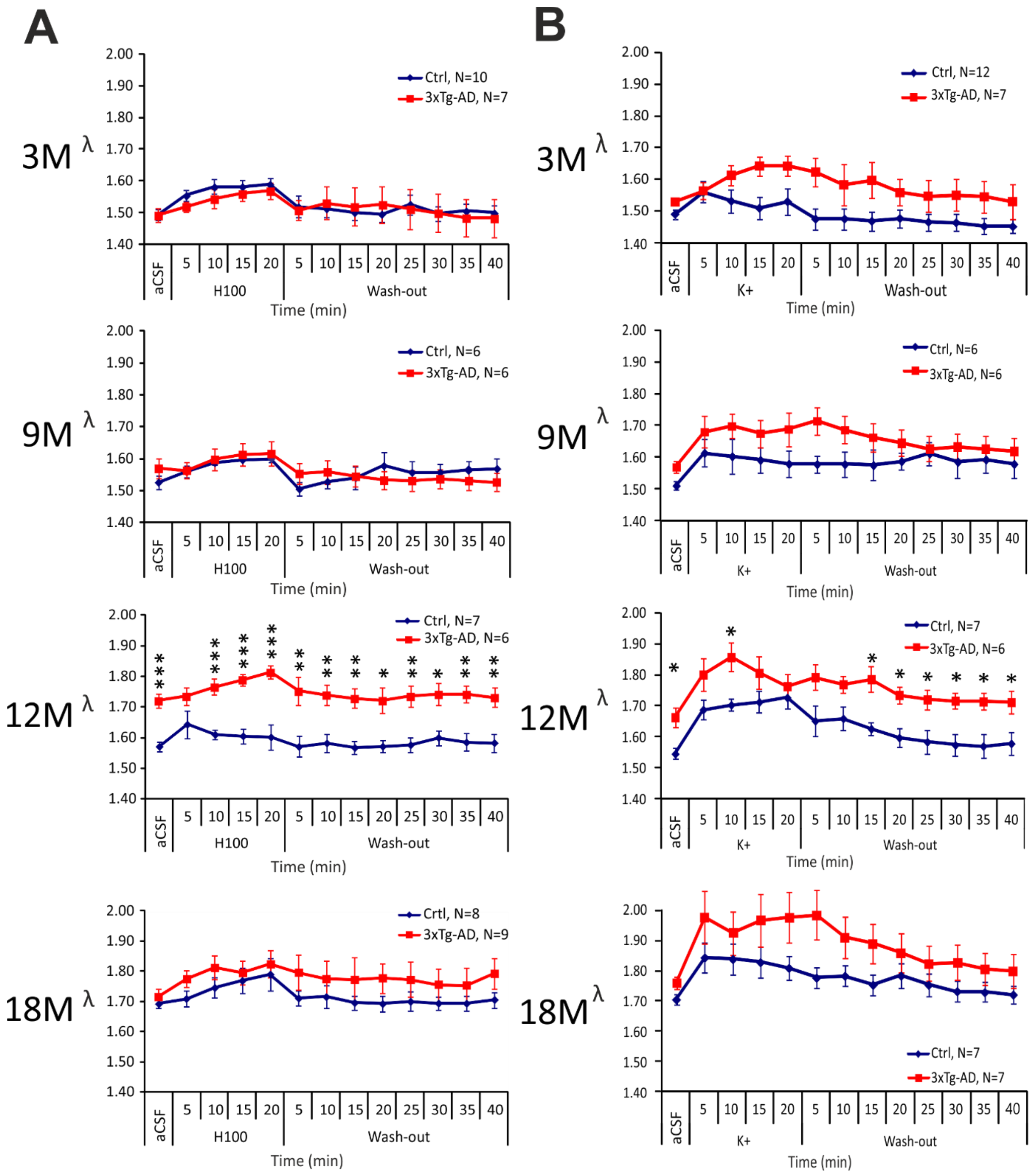

Supplementary Figure 1. Age-dependent changes of the absolute values of the tortuosity λ in control and 3xTg-AD mice. Time course of the tortuosity (λ) changes during a 20-min application of $aCSF_{H-100}$ **(A)** or $aCSF_{K+}$ **(B)** and a 40-min washout in 3-, 9-, 12- and 18-month-old control animals and age-matched 3xTg-AD mice. Data are presented as mean ± SEM. Asterisks indicate significant differences between Ctrl and 3xTg-AD mice (*p<0.05, **p<0.01, ***p<0.001). Note that the only significant differences in λ were detected in 12-month-old mice, where λ values were higher in 3xTg-AD mice than in controls during almost the entire experiment due to its larger basal value.

Ctrl – control mice; 3xTg-AD – triple transgenic model of Alzheimer's disease; 3M, 9M, 12M, 18M – 3-, 9-, 12-, 18-month-old animals; aCSF - artificial cerebrospinal fluid; H100 - hypotonic artificial cerebrospinal fluid; K+ - artificial cerebrospinal fluid with 50 mM K^+; N - number of animals.
