# Supplementary material for: Compromised Astrocyte Swelling/Volume Regulation in the Hippocampus of the Triple Transgenic Mouse Model of Alzheimer’s Disease
Source: Front Aging Neurosci. 2022 Jan 27;13:783120. doi: 10.3389/fnagi.2021.783120 (PMC8829436; doi:10.3389/fnagi.2021.783120)
Supplement: Supplementary file 2 [file Image_2.pdf]

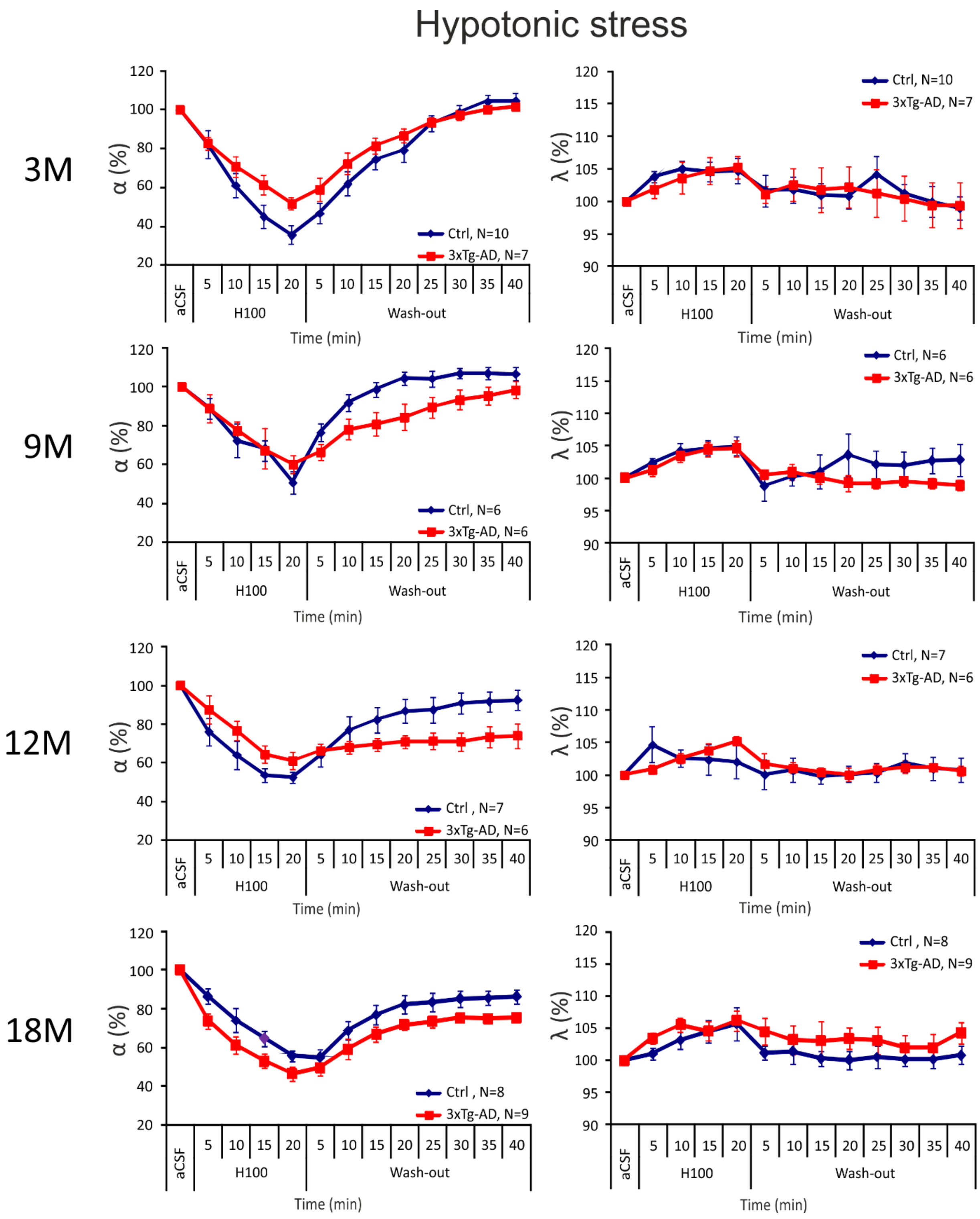

Supplementary Fig. 2. Age-dependent changes of the relative values of the ECS diffusion parameters in control and 3xTg-AD mice evoked by hypo-osmotic stress. Left: Time course of the volume fraction (α) changes during a 20-min application of $aCSF_{H-100}$ and a 40-min washout in 3-, 9-, 12- and 18-month-old control animals and age-matched 3xTg-AD mice. Right: Time course of tortuosity (λ) changes during an $aCSF_{H-100}$ application and following washout in 3-, 9-, 12- and 18-month-old control animals and age-matched 3xTg-AD mice. To estimate the relative changes, the control values were set to 100%, and the relative changes were calculated and presented as mean ±

SEM. No significant differences between controls and age-matched 3xTg-AD mice in the relative values of the ECS diffusion parameters were detected.
Ctrl – control mice; 3xTg-AD – triple transgenic model of AD; 3M, 9M, 12M, 18M – 3-, 9-, 12-, 18-month-old animals; aCSF - artificial cerebrospinal fluid; H100 - hypotonic artificial cerebrospinal fluid; N - number of animals.
