# Supplementary material for: Compromised Astrocyte Swelling/Volume Regulation in the Hippocampus of the Triple Transgenic Mouse Model of Alzheimer’s Disease
Source: Front Aging Neurosci. 2022 Jan 27;13:783120. doi: 10.3389/fnagi.2021.783120 (PMC8829436; doi:10.3389/fnagi.2021.783120)
Supplement: Supplementary file 4 [file Image_4.pdf]

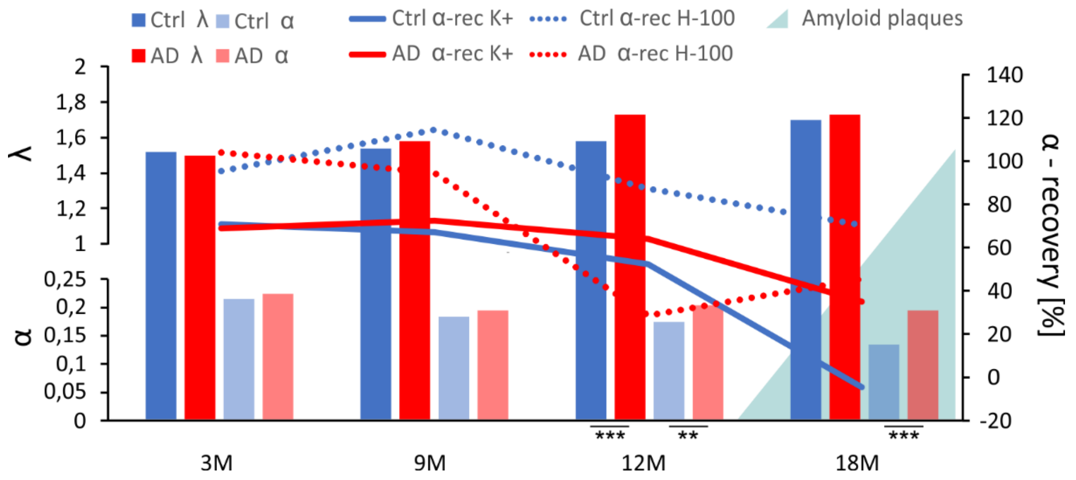

Supplementary Fig. 4. Scheme summarizing age-dependent changes in diffusion parameters of the extracellular space (ECS). Age-dependent changes in the ECS volume fraction (α) and tortuosity (λ) are shown in the bar graph. Line graphs indicate percentage volume recovery after 40-minute washout following exposure to hypoosmotic stress (H-100, dotted line) or hyperkalemia (K+, solid line).

Ctrl – control mice; 3xTg-AD – triple transgenic model of AD; 3M, 9M, 12M, 18M – 3-, 9-, 12-, 18-month-old animals; α-rec – recovery of ECS volume fraction; H100 - hypotonic artificial cerebrospinal fluid; K+ - artificial cerebrospinal fluid with 50 mM K^+.
