# Supplementary material for: Compromised Astrocyte Swelling/Volume Regulation in the Hippocampus of the Triple Transgenic Mouse Model of Alzheimer’s Disease
Source: Front Aging Neurosci. 2022 Jan 27;13:783120. doi: 10.3389/fnagi.2021.783120 (PMC8829436; doi:10.3389/fnagi.2021.783120)
Supplement: Supplementary file 5 [file Table_1.docx]

**Supplementary Table 1. Single cell RT - qPCR - List of analyzed genes**

| **Gene Name** | **NCBI RefSeq or**  **MGI RefID** | **Forward (5'-3')** | **Reverse (5'-3')** | **Size (bp)** | **Intron** | **Efficiency‎** |
| --- | --- | --- | --- | --- | --- | --- |
| *Abcc8* | NM_001357538.1 | CGAGAGTCCCTTCAATAAGCAAA | TACACTGATTCGCTGACGCT | 151 | yes | 0,97 |
| *Abcc9* | NM_001044720 | GGAAGCCTATGAAGAGCAGAAG | TCAGCCAAGTACCGGAATGT | 129 | yes | 0,99 |
| *Aldh1l1* | NM_027406.1 | CTGGAAGATGGCAAGATGATG | TTCTACCTCTGGGACATTGG | 147 | yes | 0,99 |
| *AppSwe* | [MGI: J:84847](http://www.informatics.jax.org/reference/J:84847) | TGGGTTCAAACAAAGGTGCAA | GATGAAGATCACTGTCGCTATGAC | 71 | no | 0,91 |
| *Aqp1* | NM_007472.2 | CTGGCTGCGGTATCAACC | GGATGAAGTCATAGATGAGCACTG | 132 | yes | 0,96 |
| *Aqp4* | NM_009700.2 | GATCTTTTGGACCCGCAGTTA | AGACATACTCATAAAGGGCACC | 108 | yes | 0,93 |
| *Aqp9* | NM_022026.2 | GAAGGATGGAGTGGTTCAAGTTC | TGGCACGGATACAAATGGTTT | 137 | yes | 1,00 |
| *Atp1a2* | NM_178405.3 | CCACAAGCTGTCCTTGGATG | CGTTGGGTCCATCTCTAGCC | 107 | yes | 1,06 |
| *Atp1a3* | NM_001290469.1 | CTGTCCTCGGGTTCCGTAAA | CACTAGCAGGTACCGGTTGTC | 135 | yes | 0,94 |
| *Bace1* | NM_001145947.2 | ACATTCCCAACATCTTTTCCCT | CTGCCCGTGTATAGCGAGT | 127 | yes | 0,98 |
| *Bace2* | NM_019517.5 | CCCCAGAAGGTGTTTGATGC | TGGCGTTTCAGAATTTGTCC | 120 | yes | 1,02 |
| *Best1* | NM_011913.2 | GGCTCCACCTTCAACATCAG | GCCACTCTCTTTCTTATCCGT | 87 | yes | 1,03 |
| *Chrna7* | NM_007390.3 | TCTGATTCCGTGCCCTTGAT | ACTTAGGCATTTTGCCACCA | 130 | yes | 0,99 |
| *Clcn1* | NM_001363712.1 | AGATCTTACCTGGGGGCTATG | AATTCGAAGCAAATGACGGC | 94 | yes | 0,98 |
| *Clcn2* | NM_009900.2 | TGCCAATGTCTTCCTTACTCTG | ATTCGGTAGGTGCTGCTATC | 198 | yes | 0,96 |
| *Clcn3* | NM_007711.3 | AATTGTTGACGATATTCCTGACC | TGGGGATAAACAAGCCTGAC | 151 | yes | 0,96 |
| *Clcn4* | NM_001302386.1 | ACAGACTACAACGGCTTTCC | AATTGCTCACAATGCCCTCT | 130 | yes | 0,98 |
| *Clcn7* | NM_001317404.1 | GCGGAATCCCTCAGATCAAG | TACCACAGACAGAATCACGC | 107 | yes | 0,99 |
| *Cspg4* | NM_139001.2 | ATGACCAACCCCCTGTTCT | CGAATCATTGTCTGTTCCCCT | 104 | yes | 0,96 |
| *Gabbr1* | NM_019439.3 | GCAGACTACCGAGGTCTTCA | GCTTCAGGTTTTTAACAGGCAC | 128 | yes | 0,99 |
| *Gabbr2* | NM_001081141.2 | TAGCACCATCACTCTCTGCC | GTGTGAACTGGAATCGCCTG | 98 | yes | 1,02 |
| *Gfap* | NM_001131020.1 | ATTCGCACTCAATACGAGGC | AGGTCTGCAAACTTAGACCG | 80 | yes | 0,97 |
| *Gfapδ* | NM_010277.3 | ACAGACTTTCTCCAACCTCCA | CAGGGCTCCATTTTCAATC | 159 | yes | 1,00 |
| *Gja1* | NM_010288.3 | GGTCTGAGAGCCCGAAC | CGCTCCAGTCACCCATGT | 124 | yes | 0,99 |
| *Gjb6* | NM_001010937.2 | AGGGATTTTGCAGTGACTCTT | TAGAGTGCTTGTTCACGCCA | 343 | yes | 0,96 |
| *Glul* | NM_008131.3 | CGCAAAGACCCCAACAAG | ATTCCTGCTCCATTCCAAAC | 135 | yes | 0,96 |
| *Gria1* | NM_001113325.1 | ACTCAAGCGTCCAGAATAGAAC | AATCTCAAGTCGGTAGGAATAGC | 173 | yes | 0,94 |
| *Gria2* | NM_001039195.1 | ACCAATGGGATAAGTTCGCA | TGTTCCCCACATTGATAGCAG | 120 | yes | 1,01 |
| *Gria3* | NM_016886.3 | GGTCATTCTCACGGAGGATTC | GGTGTTCTGGTTGGTGTTGTA | 123 | yes | 1,00 |
| *Gria4* | NM_001113180.1 | ACAACATTGAGACTGCCAACA | TCCAAAAATGGCAAACACCC | 83 | yes | 0,93 |
| *Grik1* | NM_146072.4 | CACGAGACGGCTGCTGAA | ACCACTGTACCTGTAGAGTTCCA | 126 | yes | 0,89 |
| *Grik2* | NM_001111268.1 | ACAATCAACAGGAACAGGACTCT | TGCTGATGAACTGTGTGAAGGA | 156 | yes | 0,92 |
| *Grik3* | NM_001081097.2 | GCTCAGAGGTGGTGGAGAATA | GCCGTGTAGGAGGAGATGAT | 166 | yes | 0,96 |
| *Grik4* | NM_175481.5 | CGCATGGTAGAATTGGAAGGT | AAGAGACTGTCAGAGATGTTGGA | 188 | yes | 0,90 |
| *Grik5* | NM_008168.2 | CCACCTTGTCCTCCGTAA | CTCCACGATACCATCCAGAT | 109 | yes | 0,89 |
| *Grin1* | NM_001177656.1 | CATCCCAAATGACAGGAAGAT | GTGGGCTTGACATACACGA | 126 | yes | 0,92 |
| *Grin2a* | NM_008170.2 | AATAAGGACCGGGAATGGGAA | GGTGGTTGTCATCTGGCTCA | 118 | yes | 1,00 |
| *Grin2b* | NM_008171.3 | GATATGCAAGCGAGAAGAGGA | AACCAGAACTTGGGAGAACAG | 149 | yes | 0,97 |
| *Grin2c* | NM_010350.2 | TTGCCATGCAGAAAGACTCC | TCGTTATGGCAGATCCCTGA | 121 | yes | 0,98 |
| *Grin2d* | NM_008172.2 | CACAGCTACATGGTGCGATA | CATAGATGAAGGCGTCCAGT | 88 | yes | 0,96 |
| *Grin3a* | NM_001033351.1 | ATGTGCTTCATGACAAGTGGT | GCACGAAGAGTCCAGAGAAA | 108 | yes | 1,00 |
| *Grin3b* | NM_130455.2 | TGGTCGGGGACAAGACATTT | TCCGTGTGGAGTGGTAGGTG | 182 | yes | 0,94 |
| *Grm1* | NM_001114333.2 | CAAAATCCAGATGAACAAAAGCG | TCACTTCCCCTTTCCGTATGA | 95 | yes | 0,95 |
| *Grm3* | NM_181850.2 | CGACCACATATTCTCAGTCCTCT | AGCACTTCGTCTAACAGCCTATA | 140 | yes | 0,96 |
| *Grm5* | NM_001081414.2 | CAGCTTAGATCGCAGCCACT | CAAGAATTTGGGTAAAATCACCA | 133 | yes | 0,91 |
| *Grm6* | NM_173372.2 | CAACTATGGTGAAAGCGGGG | TTGGGCAATACAGACACCTC | 73 | yes | 0,99 |
| *Hcn1* | NM_010408.3 | TATGAGCACCGATACCAAGG | TCGGCGTTAGCAAAAAGAGG | 137 | yes | 0,91 |
| *Hcn2* | NM_008226.2 | CTGTGGCTATTGACCGGCTA | TGCACCATCTCACGGTCATATTT | 148 | yes | 0,99 |
| *Hcn3* | NM_008227.1 | ATCCACCCCTACAGCGACTT | GATACCCACAGGCAGAACTATG | 90 | yes | 0,97 |
| *Hcn4* | NM_001081192.1 | CACTAAGATCCTCAGCCTCCT | GGTTCACGATGCGTACCAC | 125 | yes | 0,93 |
| *Itpr1* | NM_010585.5 | CCCTGACTTTGAGGAAGAATGC | GGCACGGAGACCAGAGAGTAT | 120 | yes | 0,99 |
| *Itpr2* | NM_010586.2 | GCTGACCTGGAAGTTCTGAC | GTGTTGAAATCTGGTTGATGGC | 100 | yes | 1,00 |
| *Kcnma1* | NM_001253358.1 | GTTTGTGGGCTCCATTGAGT | ATTGGCTGACAGGATAACGC | 154 | yes | 1,00 |
| *Kcnj10* | NM_001039484 | TCTGTTCATCTGTCCCGCTG | GGCTCTCTGTCTGAGTCGT | 114 | yes | 1,00 |
| *Kcnj11* | NM_010602 | TCTCTCCAGATTCCTTGTCCT | ACCATACTTCACTTTCCACG | 81 | no | 0,90 |
| *Kcnj12* | [NM_001267593.1](http://www.ncbi.nlm.nih.gov/nuccore/NM_001267593.1) | CGCAGTAGCTTGGGAAAGTT | CCTACCCCAAGAAGTCACCT | 109 | no | 0,95 |
| *Kcnj16* | NM_001252210 | CGCTTCAGCTATTTTGCCCT | CACATGGTTTGGTCGGAAGT | 87 | no | 1,00 |
| *Kcnj2* | [NM_008425.4](http://www.ncbi.nlm.nih.gov/nuccore/NM_008425.4) | GCTTCTGTAATCCCCACTTCC | TGGGGTTCTTTTGACCAGC | 116 | yes | 1,03 |
| *Kcnj4* | NM_008427.4 | GACCCTCCTCGGACCTTAC | CTGGCCGTTCTTCTTGACAA | 150 | yes | 0,96 |
| *Kcnj8* | [NM_008428.4](http://www.ncbi.nlm.nih.gov/nuccore/NM_008428.4) | GCTTACATGGAGAAAGGCACC | GAGAAACGCAGACGTGAATG | 90 | yes | 1,03 |
| *Kcnk1* | NM_008430 | GGGAAATTGGAATTGGGACTTCA | TGCCGATGACAGAGTAGATGAT | 134 | yes | 0,97 |
| *Kcnk10* | NM_001316664 | GCTGGGACAGTCATCACAAC | GCCAATAAGAAACCAAAAAGCG | 119 | yes | 0,99 |
| *Kcnk2* | NM_001281847 | CTGAGCATGATTGGGGACTG | CCTCGTTTCCTTGAACTCGG | 123 | yes | 1,01 |
| *Kcnk3* | NM_010608 | TCCTTCTACTTCGCCATCAC | ATGCAGAACACCTTGCCTC | 86 | yes | 1,00 |
| *Kcnk5* | NM_021542 | TGGTGACAGAAGAATGGAACT | GTATCGGTATAGGGCGTGGT | 128 | yes | 0,96 |
| *Kcnk9* | NM_001033876 | GCCATCACTGTCATCACAACT | CTCTGGAACATAACCAGCGTC | 122 | yes | 0,96 |
| *Lrp1* | [NM_008512.2](http://www.ncbi.nlm.nih.gov/nuccore/NM_008512.2) | CTGTATCTCAAAGGGCTGGC | GGCATCTCTGGGCTTTACTC | 101 | yes | 1,00 |
| *LRRC8A* | [NM_177725.4](http://www.ncbi.nlm.nih.gov/nuccore/NM_177725.4) | CCACAACAACCTGACCTTCC | ACATTGTTGCCCAGGTGTAG | 147 | yes | 0,96 |
| *Nes* | NM_016701.3 | AGCAACTGGCACACCTCAA | GGTATTAGGCAAGGGGGAAG | 233 | yes | 0,98 |
| *P2rx7* | NM_001038839.2 | AGACTACACCTTCCCTTTGC | TTGGAAAGATCCTCAGGACACA | 102 | yes | 1,03 |
| *P2ry1* | NM_001282016.1 | GCAGAATGGAGACACGAGTT | TTGCTTCTTCTTGACCTGTGTAT | 114 | yes | 1,04 |
| *Pdgfra* | NM_001083316.1 | AAGAGACCCTCCTTCTACCAC | TATCAGAGTCCACCCGCAT | 142 | yes | 0,97 |
| *Psen1M146* | [MGI: J:84847](http://www.informatics.jax.org/reference/J:84847) | ATCAGTGTCATTGTCGTGGTG | ACGGCGACATTGTAGGTCT | 161 | yes | 0,97 |
| *Slc12a2* | NM_009194.3 | CATCGCCGACTTCGTCATAG | AAATCCGGCCCAAAGTTCTC | 108 | yes | 1,00 |
| *Slc12a4* | NM_009195 | TACAAGTACATCGAGTACCAAGG | GTCTAACTTAAGCAGCACCAGGAG | 159 | yes | 0,95 |
| *Slc12a6* | NM_133649 | CATTCCAGGGTTGGCTAGTG | TGTGACCGAGGGAAAGAAGA | 190 | yes | 0,92 |
| *Slc1a2* | NM_011393.2 | CCATGCTCCTCATTCTCACAG | CAAAAGAATCGCCCACCACA | 120 | yes | 1,00 |
| *Slc1a3* | NM_148938.3 | AGAGCCTCACCAAGGAAGAT | TATACGGTCGGAGGGCAAAT | 114 | yes | 0,96 |
| *Slc8a1* | [NM_011406.3](http://www.ncbi.nlm.nih.gov/nuccore/NM_011406.3) | ATTGCGGAAATGGGGCG | GGGCCAGGTTCGTCTTCTTA | 118 | yes | 0,99 |
| *Slc8a2* | [NM_148946.2](http://www.ncbi.nlm.nih.gov/nuccore/NM_148946.2) | AGTTTTCTCCCCAGGTGTAGT | GAAGAGCAGTCGCTTGTCC | 106 | yes | 0,98 |
| *Slc8a3* | [NM_001167920.1](http://www.ncbi.nlm.nih.gov/nuccore/NM_001167920.1) | GTTGTTTTTGTGGCATTCGGC | CAGGAAGACATTGACGGCATTA | 129 | yes | 0,99 |
| *TauP301L* | [MGI: J:84847](http://www.informatics.jax.org/reference/J:84847) | GAGTCCAGTCGAAGATTGGG | ACTGGCGACTTGTACACGAT | 145 | yes | 0,98 |
| *Trpv2* | [NM_011706.3](http://d360prx.biomed.cas.cz:2259/nuccore/NM_011706.2) | ACCTAGTCTTCCTTTTCGGCT | ATATGGGACTGGCTCCTCCTC | 140 | yes | 0,95 |
| *Trpv4* | [NM_022017.3](http://www.ncbi.nlm.nih.gov/nuccore/NM_022017.3) | TGTTCACGAAGAAATGCCCT | ACCACCAGCACAGAGTAGAT | 88 | yes | 0,96 |
| *Vdac1* | NM_011694.4 | ACAAGAAGTTGGAGACTGCTG | AGGCTAGAGTTGTTCACTTTGG | 130 | yes | 0,98 |
| *Vdac2* | NM_011695 | GTGTATCCCTCCACCCTATGC | CGTCTTCACATCCAGCTTCAC | 97 | yes | 0,99 |
| *Vdac3* | NM_011696.1 | AGCCAAATCCAAACTGTCTCA | TGAGCCTCCAAACTCAGTGC | 103 | yes | 1,02 |
| *Vim* | NM_011701.4 | TGCCAACCTTTTCTTCCCTG | TCTCTGGTCTCAACCGTCTT | 109 | yes | 0,94 |
| *Actb* | NM_007393.4 | GCTCCTAGCACCATGAAGAT | TAAAACGCAGCTCAGTAACAG | 194 | 125 | 0,93 |
| *Ppia* | NM_008907.1 | GTTCTTCGACATCACGGC | AATCCTTTCTCTCCAGTGCTCA | 120 | 2215 | 0,95 |
| *Ywhaz* | NM_001253805.1 | AATGAAGGGTGACTACTACCG | ATGCTTCTTGGTATGCTTGCT | 98 | 666 | 0,96 |
| *Gapdh* | NM_001289726.1 | AAAATGGTGAAGGTCGGTGT | AATGAAGGGGTCGTTGATGG | 111 | 1834 | 0,92 |
| *Pgk1* | NM_008828.3 | Reference Gene Panel Mouse (A102, TATAA Biocenter) | | 170 | yes | >0.9 |
| *Hprt1* | NM_013556.2 | Reference Gene Panel Mouse (A102, TATAA Biocenter) | | 141 | yes | >0.9 |
| *Spike* |  | Universal RNA Spike II (RS25SII, TATAA Biocenter) | | 69 | no | 1,00 |
| *ValidPrime* |  | ValidPrime®(A106S25, TATAA Biocenter) | | 105 | no | 0,98 |
