# Supplementary material for: Compromised Astrocyte Swelling/Volume Regulation in the Hippocampus of the Triple Transgenic Mouse Model of Alzheimer’s Disease
Source: Front Aging Neurosci. 2022 Jan 27;13:783120. doi: 10.3389/fnagi.2021.783120 (PMC8829436; doi:10.3389/fnagi.2021.783120)
Supplement: Supplementary file 6 [file Table_2.docx]

**Supplementary Table 2. ECS diffusion parameters in control and 3xTg-AD mice evoked by hypo-osmotic stress (aCSF_H-100_)**
