# Supplementary material for: Compromised Astrocyte Swelling/Volume Regulation in the Hippocampus of the Triple Transgenic Mouse Model of Alzheimer’s Disease
Source: Front Aging Neurosci. 2022 Jan 27;13:783120. doi: 10.3389/fnagi.2021.783120 (PMC8829436; doi:10.3389/fnagi.2021.783120)
Supplement: Supplementary file 8 [file Table_4.docx]

**Supplementary Table 4. Astrocyte volume changes evoked by a 20-minute application of aCSF_H-100_**

| **3M** | | **aCSF** | **Application of aCSF_H-100_** | | | | **Washout (aCSF)** | | **n** |
| --- | --- | --- | --- | --- | --- | --- | --- | --- | --- |
|  |  | 0min | 5min | 10min | 15min | 20min | 40min | 60min |  |
| **Control** | Vc (%) | 100.00 | 111.67 | 112.86 | 117.64 | 121.15 | 106.91 | 112.21 | 38 |
|  | SEM | 0.00 | 3.05 | 2.57 | 3.10 | 3.57 | 3.38 | 3.81 |  |
| **3xTg-AD** | Vc (%) | 100.00 | 120.64 | 119.16 | 119.77 | 118.17 | 97.81 | 103.43 | 22 |
|  | SEM | 0.00 | 3.74 | 3.19 | 4.33 | 4.52 | 4.02 | 4.68 |  |
| **Two-way ANOVA** | Significance | ns | ns | ns | ns | ns | ns | ns |  |
|  | p-value | >0.9999 | 0.3941 | 0.7937 | 0.9995 | 0.9960 | 0.3768 | 0.4222 |  |

| **9M** | | **aCSF** | **Application of aCSF_H-100_** | | | | **Washout (aCSF)** | | **n** |
| --- | --- | --- | --- | --- | --- | --- | --- | --- | --- |
|  |  | 0min | 5min | 10min | 15min | 20min | 40min | 60min |  |
| **Control** | Vc (%) | 100.00 | 120.78 | 123.86 | 125.83 | 128.35 | 103.89 | 109.10 | 38 |
|  | SEM | 0.00 | 5.00 | 5.05 | 5.07 | 5.19 | 4.13 | 5.38 |  |
| **3xTg-AD** | Vc (%) | 100.00 | 111.91 | 109.46 | 108.49 | 108.36 | 92.09 | 88.48 | 38 |
|  | SEM | 0.00 | 4.22 | 3.73 | 4.05 | 3.90 | 2.10 | 2.86 |  |
| **Two-way ANOVA** | Significance | ns | ns | ns | ***** | ****** | ns | ****** |  |
|  | p-value | >0.9999 | 0.5847 | 0.0769 | 0.0162 | 0.0032 | 0.2368 | 0.0021 |  |

| **12M** | | **aCSF** | **Application of aCSF_H-100_** | | | | **Washout (aCSF)** | | **n** |
| --- | --- | --- | --- | --- | --- | --- | --- | --- | --- |
|  |  | 0min | 5min | 10min | 15min | 20min | 40min | 60min |  |
| **Control** | Vc (%) | 100.00 | 117.57 | 117.17 | 116.86 | 115.52 | 98.81 | 99.49 | 35 |
|  | SEM | 0.00 | 4.30 | 3.68 | 3.70 | 3.60 | 1.93 | 2.34 |  |
| **3xTg-AD** | Vc (%) | 100.00 | 104.46 | 108.14 | 104.18 | 101.76 | 84.85 | 86.51 | 26 |
|  | SEM | 0.00 | 6.38 | 3.91 | 4.05 | 4.64 | 3.48 | 3.98 |  |
| **Two-way ANOVA** | Significance | ns | ns | ns | ns | ***** | ***** | ns |  |
|  | p-value | >0.9999 | 0.0710 | 0.4303 | 0.0888 | 0.0496 | 0.0442 | 0.0762 |  |
| **18M** | | **aCSF** | **Application of aCSF_H-100_** | | | | **Washout (aCSF)** | | **n** |
|  |  | 0min | 5min | 10min | 15min | 20min | 40min | 60min |  |
| **Control** | Vc (%) | 100.00 | 117.72 | 112.86 | 112.12 | 109.79 | 93.14 | 89.75 | 35 |
|  | SEM | 0.00 | 4.26 | 4.85 | 5.17 | 5.42 | 3.04 | 3.20 |  |
| **3xTg-AD** | Vc (%) | 100.00 | 121.04 | 117.56 | 110.81 | 109.12 | 86.83 | 85.51 | 26 |
|  | SEM | 0.00 | 6.44 | 7.00 | 6.31 | 5.99 | 4.25 | 3.67 |  |
| **Two-way ANOVA** | Significance | ns | ns | ns | ns | ns | ns | ns |  |
|  | p-value | >0.9999 | 0.9988 | 0.9892 | >0.9999 | >0.9999 | 0.9451 | 0.9941 |  |

**Significancy of differences between age groups – Two-way ANOVA**

| **Control** | 5min | | 10min | | 15min | | 20min | | 40min | | 60min | |
| --- | --- | --- | --- | --- | --- | --- | --- | --- | --- | --- | --- | --- |
|  | Signif. | p-value | Signif. | p-value | Signif. | p-value | Signif. | p-value | Signif. | p-value | Signif. | p-value |
| 3M vs. 9M | ns | 0.3049 | ns | 0.1553 | ns | 0.4018 | ns | 0.5174 | ns | 0.9392 | ns | 0.9346 |
| 3M vs. 12M | ns | 0.6887 | ns | 0.8533 | ns | 0.9989 | ns | 0.7197 | ns | 0.4309 | ns | 0.0830 |
| 3M vs. 18M | ns | 0.6712 | ns | >0.9999 | ns | 0.7318 | ns | 0.1475 | ns | 0.0505 | ******* | 0.0002 |
| 9M vs. 12M | ns | 0.9322 | ns | 0.5955 | ns | 0.3386 | ns | 0.0789 | ns | 0.7792 | ns | 0.2770 |
| 9M vs. 18M | ns | 0.9406 | ns | 0.1696 | ns | 0.0520 | ****** | 0.0031 | ns | 0.1865 | ****** | 0.0018 |
| 12M vs. 18M | ns | >0.9999 | ns | 0.8602 | ns | 0.8216 | ns | 0.7213 | ns | 0.7275 | ns | 0.2835 |

| **3xTg-AD** | 5min | | 10min | | 15min | | 20min | | 40min | | 60min | |
| --- | --- | --- | --- | --- | --- | --- | --- | --- | --- | --- | --- | --- |
|  | Signif. | p-value | Signif. | p-value | Signif. | p-value | Signif. | p-value | Signif. | p-value | Signif. | p-value |
| 3M vs. 9M | ns | 0.4431 | ns | 0.3479 | ns | 0.2175 | ns | 0.3378 | ns | 0.7621 | ns | 0.0530 |
| 3M vs. 12M | ns | 0.0527 | ns | 0.3034 | ns | 0.0668 | ns | 0.0479 | ***** | 0.1719 | ***** | 0.0385 |
| 3M vs. 18M | ns | >0.9999 | ns | 0.9944 | ns | 0.4901 | ns | 0.4815 | ns | 0.3064 | ***** | 0.0246 |
| 9M vs. 12M | ns | 0.5392 | ns | 0.9953 | ns | 0.8662 | ns | 0.6360 | ns | 0.5629 | ns | 0.9848 |
| 9M vs. 18M | ns | 0.3561 | ns | 0.4648 | ns | 0.9755 | ns | 0.9991 | ns | 0.7805 | ns | 0.9505 |
| 12M vs. 18M | ***** | 0.0324 | ns | 0.4056 | ns | 0.6936 | ns | 0.6186 | ns | 0.9880 | ns | 0.9984 |
