# Supplementary material for: Compromised Astrocyte Swelling/Volume Regulation in the Hippocampus of the Triple Transgenic Mouse Model of Alzheimer’s Disease
Source: Front Aging Neurosci. 2022 Jan 27;13:783120. doi: 10.3389/fnagi.2021.783120 (PMC8829436; doi:10.3389/fnagi.2021.783120)
Supplement: Supplementary file 9 [file Table_5.docx]

**Supplementary Table 5. Astrocyte volume changes evoked by a 20-minute application of aCSF_K+_**

| **3M** | | **aCSF** | **Application of aCSF_K+_** | | | | **Washout (aCSF)** | | **n** |
| --- | --- | --- | --- | --- | --- | --- | --- | --- | --- |
|  |  | 0 min | 5 min | 10 min | 15 min | 20 min | 40 min | 60 min |  |
| **Control** | Vc (%) | 100.00 | 161.00 | 167.31 | 169.07 | 168.48 | 111.16 | 115.25 | 15 |
|  | SEM | 0.00 | 5.12 | 5.38 | 6.21 | 7.95 | 10.52 | 12.83 |  |
| **3xTg-AD** | Vc (%) | 100.00 | 122.74 | 128.48 | 127.74 | 127.15 | 85.62 | 82.94 | 20 |
|  | SEM | 0.00 | 4.97 | 4.16 | 4.74 | 5.33 | 5.52 | 4.81 |  |
| **Two-way ANOVA** | Significance | ns | ******* | ******* | ******* | ******* | ***** | ****** |  |
|  | p-value | >0.9999 | <0.0001 | <0.0001 | <0.0001 | <0.0001 | 0.0228 | 0.0015 |  |

| **9M** | | **aCSF** | **Application of aCSF_K+_** | | | | **Washout (aCSF)** | | **n** |
| --- | --- | --- | --- | --- | --- | --- | --- | --- | --- |
|  |  | 0 min | 5 min | 10 min | 15 min | 20 min | 40 min | 60 min |  |
| **Control** | Vc (%) | 100.00 | 130.87 | 133.19 | 133.02 | 130.86 | 87.00 | 80.85 | 15 |
|  | SEM | 0.00 | 4.62 | 5.36 | 4.85 | 5.18 | 2.96 | 5.28 |  |
| **3xTg-AD** | Vc (%) | 100.00 | 119.24 | 123.75 | 119.61 | 116.67 | 83.37 | 83.25 | 33 |
|  | SEM | 0.00 | 3.58 | 3.92 | 4.08 | 4.52 | 3.09 | 3.63 |  |
| **Two-way ANOVA** | Significance | ns | ns | ns | ns | ns | ns | ns |  |
|  | p-value | >0.9999 | 0.3290 | 0.5898 | 0.1767 | 0.1296 | 0.9962 | 0.9997 |  |

| **12M** | | **aCSF** | **Application of aCSF_K+_** | | | | **Washout (aCSF)** | | **n** |
| --- | --- | --- | --- | --- | --- | --- | --- | --- | --- |
|  |  | 0 min | 5 min | 10 min | 15 min | 20 min | 40 min | 60 min |  |
| **Control** | Vc (%) | 100.00 | 158.92 | 164.22 | 162.10 | 163.24 | 95.43 | 93.16 | 15 |
|  | SEM | 0.00 | 7.80 | 9.34 | 8.60 | 9.40 | 5.26 | 4.62 |  |
| **3xTg-AD** | Vc (%) | 100.00 | 117.21 | 114.72 | 112.19 | 109.14 | 80.00 | 78.28 | 25 |
|  | SEM | 0.00 | 4.17 | 5.12 | 5.65 | 6.14 | 4.06 | 3.30 |  |
| **Two-way ANOVA** | Significance | ns | ******* | ******* | ******* | ******* | ns | ns |  |
|  | p-value | >0.9999 | <0.0001 | <0.0001 | <0.0001 | <0.0001 | 0.3269 | 0.3713 |  |

| **18M** | | **aCSF** | **Application of aCSF_K+_** | | | | **Washout (aCSF)** | | **n** |
| --- | --- | --- | --- | --- | --- | --- | --- | --- | --- |
|  |  | 0 min | 5 min | 10 min | 15 min | 20 min | 40 min | 60 min |  |
| **Control** | Vc (%) | 127.62 | 131.65 | 136.56 | 132.26 | 93.12 | 91.10 | 127.62 | 16 |
|  | SEM | 5.70 | 5.29 | 5.58 | 5.81 | 5.86 | 5.52 | 5.70 |  |
| **3xTg-AD** | Vc (%) | 128.83 | 131.34 | 128.27 | 125.46 | 75.42 | 72.53 | 128.83 | 22 |
|  | SEM | 5.06 | 5.63 | 5.76 | 6.17 | 3.64 | 3.82 | 5.06 |  |
| **Two-way ANOVA** | Significance | ns | ns | ns | ns | ns | ns | ns |  |
|  | p-value | >0.9999 | >0.9999 | >0.9999 | 0.8597 | 0.9451 | 0.0900 | 0.0647 |  |

**Significancy of differences between age groups – Two-way ANOVA**

| **Controls** | 5min | | 10min | | 15min | | 20min | | 40min | | 60min | |
| --- | --- | --- | --- | --- | --- | --- | --- | --- | --- | --- | --- | --- |
|  | Signif. | p-value | Signif. | p-value | Signif. | p-value | Signif. | p-value | Signif. | p-value | Signif. | p-value |
| 3M vs. 9M | ****** | 0.0047 | ******* | 0.0009 | ******* | 0.0004 | ******* | 0.0002 | ***** | 0.0368 | ******* | 0.0008 |
| 3M vs. 12M | ns | 0.9956 | ns | 0.9860 | ns | 0.8652 | ns | 0.9367 | ns | 0.2975 | ns | 0.0673 |
| 3M vs. 18M | ****** | 0.0010 | ******* | 0.0004 | ****** | 0.0015 | ******* | 0.0003 | ns | 0.1738 | ***** | 0.0328 |
| 9M vs. 12M | ***** | 0.0102 | ****** | 0.0033 | ****** | 0.0070 | ****** | 0.0020 | ns | 0.7831 | ns | 0.5178 |
| 9M vs. 18M | ns | 0.9829 | ns | 0.9981 | ns | 0.9781 | ns | 0.9986 | ns | 0.8996 | ns | 0.6520 |
| 12M vs. 18M | ****** | 0.0025 | ****** | 0.0014 | ***** | 0.0208 | ****** | 0.0028 | ns | 0.9937 | ns | 0.9955 |

| **3xTg-AD** | 5min | | 10min | | 15min | | 20min | | 40min | | 60min | |
| --- | --- | --- | --- | --- | --- | --- | --- | --- | --- | --- | --- | --- |
|  | Signif. | p-value | Signif. | p-value | Signif. | p-value | Signif. | p-value | Signif. | p-value | Signif. | p-value |
| 3M vs. 9M | ns | 0.9381 | ns | 0.8616 | ns | 0.5339 | ns | 0.3056 | ns | 0.9823 | ns | >0.9999 |
| 3M vs. 12M | ns | 0.8232 | ns | 0.1375 | ns | 0.0720 | ***** | 0.0255 | ns | 0.8162 | ns | 0.8853 |
| 3M vs. 18M | ns | 0.7916 | ns | 0.9726 | ns | 0.9998 | ns | 0.9940 | ns | 0.4088 | ns | 0.3903 |
| 9M vs. 12M | ns | 0.9842 | ns | 0.3802 | ns | 0.5544 | ns | 0.5420 | ns | 0.9335 | ns | 0.8152 |
| 9M vs. 18M | ns | 0.3596 | ns | 0.5668 | ns | 0.4516 | ns | 0.4387 | ns | 0.5281 | ns | 0.2614 |
| 12M vs. 18M | ns | 0.2444 | ***** | 0.0389 | ***** | 0.0490 | ***** | 0.0443 | ns | 0.8826 | ns | 0.7930 |
